# Bone marrow adipocytes promote the Warburg phenotype in metastatic prostate tumors *via* HIF-1α activation

**DOI:** 10.18632/oncotarget.11712

**Published:** 2016-08-30

**Authors:** Jonathan D. Diedrich, Erandi Rajagurubandara, Mackenzie K. Herroon, Gargi Mahapatra, Maik Hüttemann, Izabela Podgorski

**Affiliations:** ^1^ Department of Pharmacology, Wayne State University School of Medicine, Detroit, MI, USA; ^2^ Center for Molecular Medicine and Genetics, Wayne State University School of Medicine, Detroit, MI, USA; ^3^ Karmanos Cancer Institute, Wayne State University School of Medicine, Detroit, MI, USA

**Keywords:** bone marrow adipocytes, bone metastasis, prostate cancer, glycolysis, Warburg effect

## Abstract

Metabolic adaptation is increasingly recognized as a key factor in tumor progression, yet its involvement in metastatic bone disease is not understood. Bone is as an adipocyte-rich organ, and a major site of metastasis from prostate cancer. Bone marrow adipocytes are metabolically active cells capable of shaping tumor metabolism *via* lipolysis and lipid transfer. In this study, using *in vitro* and *in vivo* models of marrow adiposity, we demonstrate that marrow fat cells promote Warburg phenotype in metastatic prostate cancer cells. We show increased expression of glycolytic enzymes, increased lactate production, and decreased mitochondrial oxidative phosphorylation in tumor cells exposed to adipocytes that require paracrine signaling between the two cell types. We also reveal that prostate cancer cells are capable of inducing adipocyte lipolysis as a postulated mechanism of sustenance. We provide evidence that adipocytes drive metabolic reprogramming of tumor cells *via* oxygen-independent mechanism of HIF-1α activation that can be reversed by HIF-1α downregulation. Importantly, we also demonstrate that the observed metabolic signature in tumor cells exposed to adipocytes mimics the expression patterns seen in patients with metastatic disease. Together, our data provide evidence for a functional relationship between marrow adipocytes and tumor cells in bone that has likely implications for tumor growth and survival within the metastatic niche.

## INTRODUCTION

Altered metabolic phenotype and the ability to adapt and thrive in harsh microenvironments are features that distinguish cancer cells from normal cells [1, 2]. It is well-accepted that most tumor cells rely on accelerated glucose metabolism for support of anabolic processes such as lipid, protein and nucleic acid syntheses, and, consequently, for growth and survival [3, 4]. This phenomenon, known as the “Warburg Effect”, is one of the hallmarks of cancer, and the glycolytic fueling of growth is thought to be the key feature behind the progression of most tumors [5]. However, it is becoming increasingly apparent that the metabolic phenotype of a cancer cell can vary depending on the tumor type and the stage of the disease. The possession of a distinct metabolic phenotype is especially evident in primary prostate cancers, which unlike other solid tumors do not undergo the classical “glycolytic switch” [6, 7]. Instead, these tumors generally exhibit activation of β-oxidation pathways as the means of supporting tumor cell viability under conditions of energy stress [8-11]. Primary prostate cancer cells have unique abilities to exploit fatty acid metabolic pathways to foster malignant transformation. The uptake of lipids from the microenvironment, aberrant *de novo* lipid synthesis and alterations in fatty acid catabolism and steroidogenesis pathways are now emerging as key mechanisms linking dysregulated lipid metabolism in the primary prostate tumor with subsequent progression and reduced survival [7, 12, 13]. In contrast to the primary disease, however, the metabolic phenotype of metastatic prostate cancers is not well-understood. The acquisition of a glycolytic phenotype in advanced stages of prostate cancer has been suggested by the reports of increased accumulation of fluorodeoxyglucose (FDG) [14] and the immunohistochemical evidence of expression of glycolytic markers and monocarboxylate transporters [15]. The mechanisms contributing to metabolic adaptation and progression of metastatic prostate tumors in bone has not, however, been previously explored and are not known.

Metastatic growth in bone is a complex process involving reciprocal interactions between the tumor cells and the host bone microenvironment. One of the most abundant, yet overlooked components of the metastatic marrow niche are the bone marrow adipocytes [16-18]. Adipocyte numbers in the marrow increase with age, obesity and metabolic disorders [18-23], all of which are also risk factors for metastatic disease [24-28]. We and others have shown previously that marrow fat cells, as highly metabolically active cells, can serve as a source of lipids for cancer cells, and promote growth, invasion, and aggressiveness of metastatic tumors in bone [16, 29, 30]. Based on the growing evidence from cancers that grow in adipocyte-rich tissues, it is becoming apparent that one way adipocytes can affect tumor cell behavior is through modulation of cancer cell metabolism [31]. Although direct effects of adipocyte-supplied lipids on tumor metabolism have not been investigated in the context of metastatic prostate cancer, there have been studies in other cancers demonstrating that some lipids do have the ability to enhance the Warburg Effect in tumor cells [32-36]. Reciprocally, tumor cells have been shown to act as metabolic parasites by inducing lipolysis in adipocytes [37, 38]. This is important in the regulation of tumor metabolism as the lipolysis-generated glycerol can feed into the glycolytic pathway [39-41] and the released fatty acids can be oxidized through β-oxidation [42, 43]. As active and vital components of the bone-tumor microenvironment, adipocytes are likely to be involved in the metabolic adaptation of tumors in the metastatic niche; however, the concept of metabolic coupling between marrow adipocytes and tumor cells leading to metabolic reprogramming in the tumor has not been explored before.

One of the principal mechanisms behind metabolic reprogramming is hypoxic stress and activation of hypoxia inducible factor (HIF) [44]. HIF-1 stimulates the conversion of glucose to pyruvate and lactate by upregulating key enzymes involved in glucose transport, glycolysis, and lactate extrusion, and by decreasing conversion of pyruvate to acetyl-CoA through transactivation of pyruvate dehydrogenase kinase (PDK1) and subsequent inhibition of pyruvate dehydrogenase (PDH) [44]. Regulation of lactate dehydrogenase (LDHa) and PDK1 by HIF-1 keeps the pyruvate away from mitochondria, thus depressing mitochondrial respiration [4]. Under normoxic conditions, HIF-1 is rapidly degraded by the ubiquitin-proteasome pathway [45]. Decreased oxygen availability prevents HIF-1 hydroxylation leading to its stabilization and activation of downstream pathways [2]. In cancer cells, HIF-1 stabilization and activation can occur during normoxia *via* multiple oxygen-independent pathways [46]. This phenomenon, termed “pseudohypoxia”, is thought to facilitate adaptation of tumor cells to harsh conditions and to promote survival and resistance to therapy [47-49]. Whether HIF-1-dependent signaling plays a role in metabolic reprogramming of prostate tumor cells in bone is not known.

The objective of this study was to elucidate the role of bone marrow adiposity in the modulation of tumor metabolism and adaptation within the bone microenvironment. Using *in vivo* models of diet-induced marrow adiposity in combination with *in vitro* models of paracrine, autocrine, and endocrine signaling between bone marrow adipocytes and prostate cancer cells, we show that bone marrow adipocytes are responsible for enhancing the glycolytic phenotype of metastatic prostate cancer cells. We demonstrate that bidirectional interaction between adipocytes and tumor cells leads to increased expression of glycolytic enzymes, increased lactate production, and decreased mitochondrial oxidative phosphorylation in tumor cells *via* necessary cancer cell-initiated paracrine crosstalk. We also reveal that the observed metabolic signature in tumor cells exposed to adipocytes mimics the expression patterns seen in patients with metastatic disease. These results offer potential mechanisms underlying the metabolic adaptation of metastatic tumors in bone and implicate bone marrow adipocytes, a cell type abundantly present in the skeleton, especially in advanced age and obesity, as viable culprits in the progression of this currently incurable disease.

## RESULTS

### *In silico* analysis of glycolysis-associated genes in prostate cancer patients

**Figure 1:**
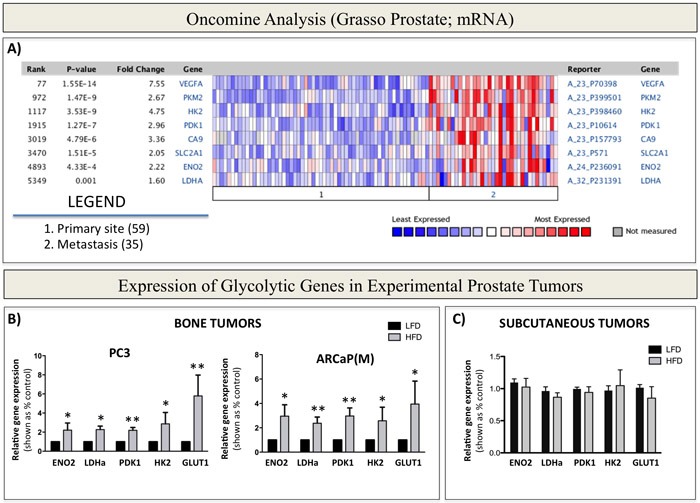
Warburg Effect-associated genes are upregulated in patients with metastatic prostate cancer and in bone tumors from mice with enhanced marrow adiposity **A.** Oncomine gene analysis comparing the expression of metabolic genes [*VEGFA, PKM2, HK2, PDK1, CA9, SLC2A1 (GLUT1), ENO2, LDHA*] in patient samples collected from metastatic or primary sites. Data were ordered by ‘overexpression’ and the threshold was adjusted to *P*-value < 1E^−^4; fold change, 2 and gene rank, top 10%. **B.** Taqman RT-PCR (Life Technologies) analysis of expression of Warburg Effect-associated genes *ENO2*, *LDHa*, *PDK1, HK2* and *GLUT1* in PC3 (left) and ARCaP(M) (right) bone tumors or **C.** subcutaneous tumors from LFD- and HFD-fed mice. Data were normalized to human *EPCAM* and represent a mean of a minimum of 3 mice/group ± SD. Values * *P* < 0.05; ** *P* < 0.01 are considered statistically significant.

The metabolic phenotype of primary prostate tumors has been well-described [8-11]; however, its characteristics in relation to the glycolytic pathway at the metastatic site are not well-understood. Therefore, we first performed an Oncomine analysis of primary and metastatic prostate tumors and compared mRNA expression of genes that encode for enzymes/proteins known to be involved in different aspects of glucose metabolism and Warburg metabolism. Specifically, thirteen available Oncomine datasets were examined for the expression of genes covering a broad spectrum of metabolic responses and associated with glucose transport [*glucose transporter 1* (*GLUT^1^*)], glycolysis [*hexokinase 2* (*HK^2^*) and *enolase 2* (*ENO^2^*)], Warburg metabolism [*pyruvate dehydrogenase kinase 1* (*PDK^1^*) and *lactate dehydrogenase* (*LDHa*)], and hypoxia [*carbonic anhydrase 9* (*CA^9^*) and *vascular endothelial growth factor* (*VEGF*)]. Our analyses revealed significant differences in the metabolic phenotype between primary and secondary sites observable in several prostate cancer datasets (Supplementary Table 1). The Grasso Prostate dataset, which contains most metastatic samples, showed the most significant upward changes in the expression of *PDK1, ENO2, HK2, GLUT1,* and *LDHa* (Figure 1A), as well as many other genes associated with the glycolysis pathway (Supplementary Figure 1 and Supplementary Table 1). Additional analyses of prostate datasets available through cbioportal.com revealed that copy number alterations/mutations/deletions in these genes are infrequent in prostate cancer (Supplementary Figure 2), pointing to the mRNA overexpression as the main mechanism behind the acquisition of metabolic phenotype. In addition to glycolytic markers, HIF-1 target genes, *CA9* and *VEGF* were also significantly upregulated in metastatic tissue (Figure 1A, Supplementary Figure 1, and Supplementary Table 2). Since HIF-1 is well-known to regulate glycolysis [44], these results further underscored the apparent metabolic differences between primary and secondary prostate cancer and prompted us to investigate the contribution of the metastatic environment to the tumor metabolic phenotype in bone.

### Bone marrow adiposity contributes to the *in vivo* glycolytic phenotype in prostate bone tumors

One important cell type credited with the ability to alter tumor metabolism is the adipocyte, whose effects on the phenotype of a tumor cell have been predominantly reported for colorectal and ovarian cancers [32, 38, 50]. Given the abundance of adipocytes in bone marrow, we hypothesized that they are likely to have similar metabolism-modulating effects on metastatic prostate cancer cells. To study effects of marrow fat cells on prostate tumor growth and progression in bone, we utilized a well-documented approach of inducing marrow adiposity with high fat diet (HFD) [18, 29, 51, 52]. We have shown previously that intratibial implantation of prostate cancer cells into this model results in accelerated tumor growth and extensive bone destruction, suggesting potential tumor-supportive effects of marrow adipocytes [29, 53]. To determine whether this adiposity-driven tumor progression in bone is associated with an altered metabolic phenotype, we analyzed mRNA expression of glycolysis-associated genes in intratibial PC3 and ARCaP(M) tumors from low fat diet (LFD) and HFD mice using human-specific Taqman probes. Our results revealed significantly increased transcript levels of *PDK1, ENO2, HK2, GLUT1,* and *LDHa* in tumors grown under conditions of HFD-induced marrow adiposity (Figure 1B), whereas the levels of mitochondrial enzymes *citrate synthase* (*CS*) and *isocitrate dehydrogenase 2* (*IDH2*), remained unaffected by diet-induced marrow adiposity (Supplementary Figure 3). Notably, this enhanced glycolytic phenotype was also observed in bone tumors from mice, in which marrow adiposity was induced with HFD, but the animals were switched to LFD upon tumor implantation into the tibia (Supplementary Figure 4). This approach allowed for tumor growth in adipocyte-rich marrow without systemic effects of HFD and revealed that expression of Warburg genes in tumor cells does not appear to be a direct effect of HFD. Furthermore, in contrast to bone tumors, expression of glycolysis-associated genes was not significantly altered in subcutaneous tumors from HFD mice in comparison to LFD mice (Figure 1C), despite the fact that HFD enhanced the growth and progression of these tumors, as we have demonstrated previously [29]. Collectively, these findings suggest that Warburg metabolism might be especially important for prostate tumor progression in bone and implicate marrow adiposity as a potential regulator of metabolic adaptation in the skeleton.

### Bone marrow adipocytes alter the metabolism of prostate cancer cells *in vitro*

**Figure 2:**
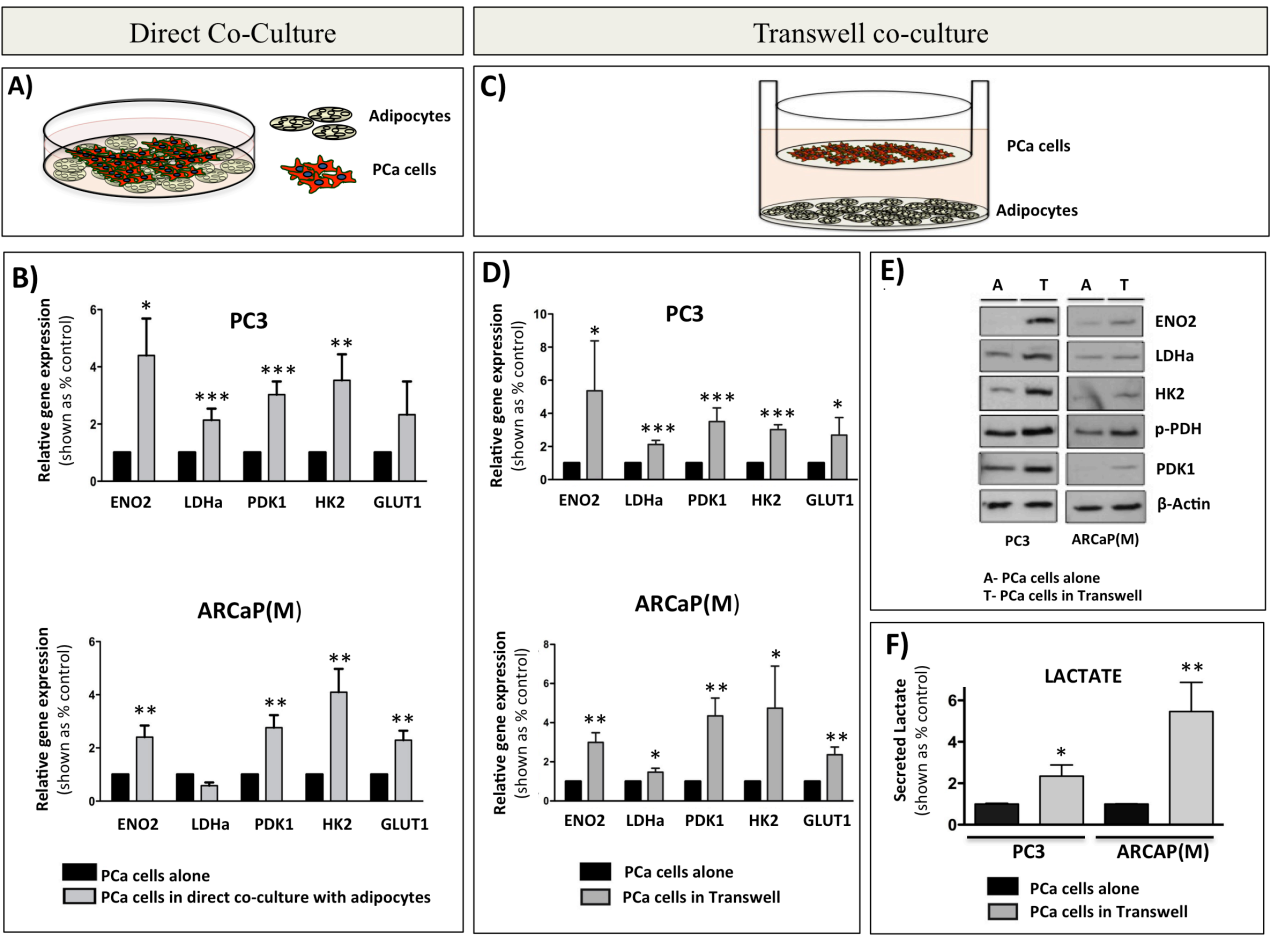
Bone marrow adipocytes enhance a glycolytic phenotype of prostate cancer cells in direct co-culture and in transwell co-culture *in vitro.* **A.** Schematic representation of a direct co-culture of tumor cells and bone marrow adipocytes. **B.** Taqman RT-PCR analysis of *ENO2, LDHa, PDK1, HK2*, and *GLUT1* expression in PC3 (Top) and ARCaP(M) (Bottom) cells cultured directly with bone marrow adipocytes. Data are normalized to *HPRT1* and shown relative to control. **C.** Schematic representation of transwell co-cultures of tumor cells and bone marrow adipocytes. **D.** Taqman RT-PCR of *ENO2, LDHa, PDK1, HK2*, and *GLUT1* expression in PC3 (top) and ARCaP(M) (bottom) in transwell co-culture. **E.** Western blot for ENO2, LDHa, HK2, phospho-PDH, and PDK1 in PC3 (left) and ARCaP(M) (right) exposed to bone marrow adipocytes in transwell co-culture. Beta-actin was used as loading control. **F.** Analysis of lactate secreted (Abcam) by PC3 (left) and ARCaP(M) (right) cells exposed to bone marrow adipocytes in transwell co-culture. Results represent a mean of at least 3 independent experiments ± SD. Values * *P* < 0.05; ** *P* < 0.01, *** *P* < 0.001 are considered statistically significant.

To determine if the glycolytic phenotype observed in intratibial tumors *in vivo* is indeed a direct effect of bone marrow adipocytes and to specifically investigate the mechanisms behind this metabolic regulation, we utilized *in vitro* models of tumor cell-adipocyte interactions. First, using human-specific Taqman RT PCR probes, we examined the expression of glycolytic markers *ENO2*, *LDHa*, *PDK1, HK2*, and GLUT1 in PC3 and ARCaP(M) cells grown in direct contact co-culture with bone marrow adipocytes (Figure 2A). Transcript levels of nearly all investigated markers were significantly increased in tumor cells grown in co-culture as opposed to those cultured alone (Figure 2B). Next, to determine if this change in metabolic phenotype requires direct interaction with adipocytes, we employed a transwell system in which adipocytes were differentiated in the bottom chamber and tumor cells were then plated on top of the insert and cultured together for 48 hours. This allowed the two cell types to share the media without direct interaction (Figure 2C). Mirroring the findings from the direct co-culture, gene expression of *ENO2*, *LDHa*, *PDK1, HK2*, and *GLUT1* was significantly increased in both PCa cell lines co-cultured with marrow adipocytes (Figure 2D). Notably, PCa cells grown in transwell co-culture with bone marrow stromal cells that were not induced to differentiate into adipocytes had no effect on the expression of glycolysis-associated genes (Supplementary Figure 5), suggesting that this observed metabolic switch in tumor cells is indeed adipocyte-driven. This enhancement of a glycolytic phenotype upon interaction with adipocytes was confirmed by the marked increases in the protein expression of ENO2, LDHa, PDK1, and HK2 (Figure 2E). We also observed increased levels of phosphorylated pyruvate dehydrogenase (p-PDH) in cells grown in transwell co-culture, which indicates elevated PDK1 activity and a shift in glucose metabolism from pyruvate to lactate (Figure 2E). To test this functionally we performed lactate analyses of media conditioned by the tumor cells in the absence or presence of adipocytes as a conventional, well-accepted approach for measuring extracellular acidification and glycolytic shift [54, 55]. Our results revealed significant increases in lactate secretion by the tumor cells exposed to adipocytes (Figure 2F), while contribution of adipocytes to lactate secretion was not significant (data not shown). This provided further evidence of acquired Warburg phenotype in tumor cells exposed to adipocytes. We also observed an augmented expression of glycolytic genes in other prostate cell lines (i.e., DU145 and C4-2B) grown in transwell co-culture with fat cells (Supplementary Figure 6), confirming the important contribution of marrow adipocytes to the metabolic phenotype of prostate tumors in bone.

**Figure 3:**
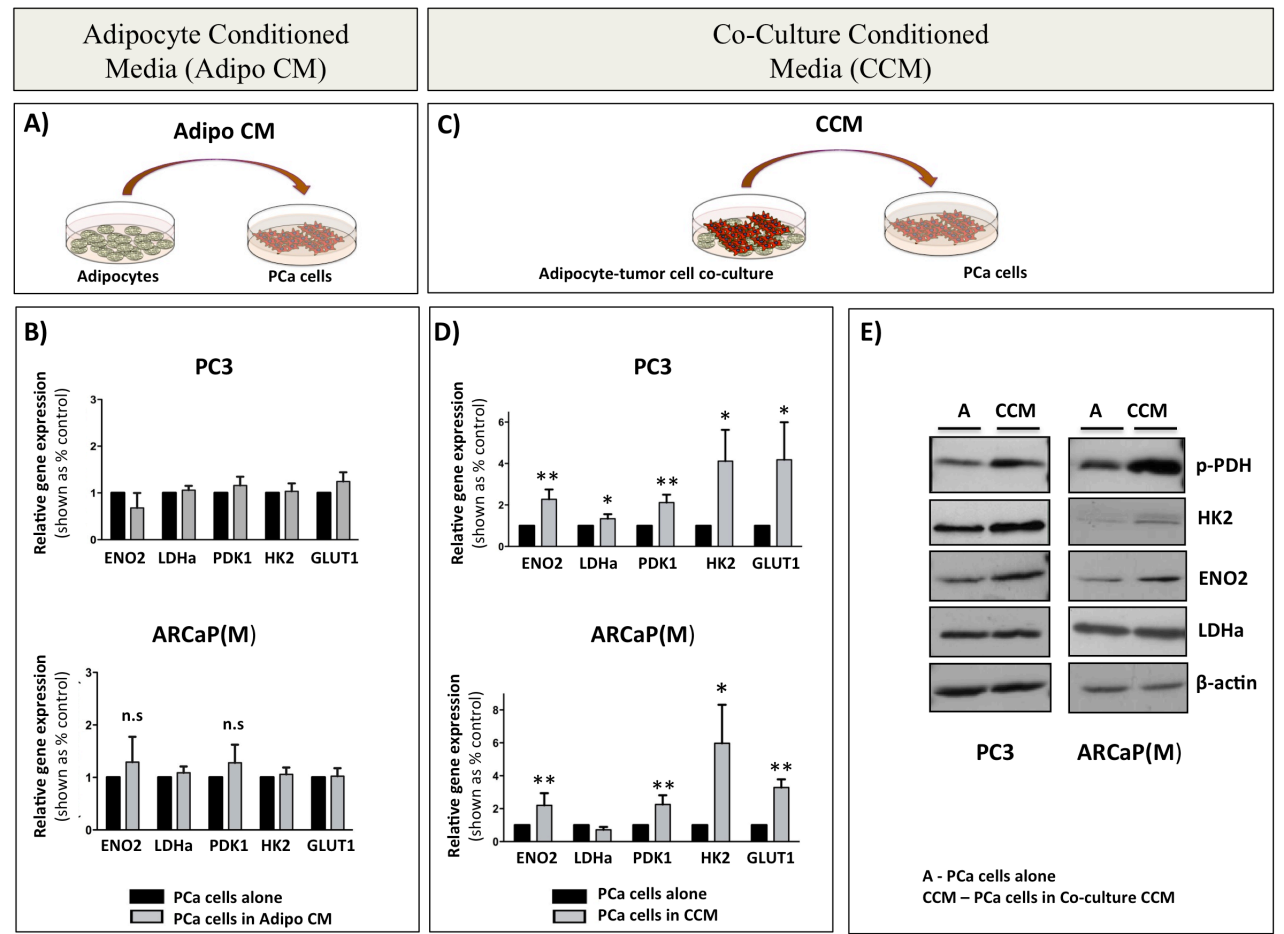
Paracrine signaling between PCa cells and bone marrow adipocytes is required for the induction of glycolytic gene and protein expression in PCa cells **A.** Schematic representation of tumor cells treated with media conditioned by bone marrow adipocytes (Adipo CM). **B.** Taqman RT-PCR analysis of *ENO2, LDHa, PDK1, HK2*, and *GLUT1* in PC3 (top) and ARCaP(M) (bottom) cells in the presence or absence of Adipo CM. Data are normalized to *HPRT1* and shown as increase relative to control. **C.** Schematic representation of tumor cell- adipocyte co-culture system (CCM). **D.** Taqman RT PCR analysis of mRNA expression of *ENO2, LDHa, PDK1, HK2*, and *GLUT1* in PC3 (top) and ARCaP(M) (bottom) cells in the presence of CCM. **E.** Western blot analysis of ENO2, LDHa, HK2, and phospho-PDH in PC3 (left) and ARCaP(M) (right) in the presence of CCM. Beta-actin was used as a loading control (bottom). Results represent a mean of at least 3 independent experiments ± SD. Values * *P* < 0.05; ** *P* < 0.01 are considered statistically significant.

The fact that both direct and transwell co-culture with adipocytes induced a glycolytic phenotype in tumor cells suggested that this process does not require physical interaction between the tumor cells and adipocytes. Therefore, we next examined whether the media conditioned by the marrow adipocytes alone (Adipo CM; Figure 3A) can bring on similar metabolic changes in tumor cells as observed in transwell co-culture. Interestingly, no changes in the mRNA expression of *ENO2*, *LDHa*, *PDK1, HK2,* and *GLUT1* were observed in either of the PCa cell lines in response to Adipo CM (Figure 3B). However, when the adipocytes were directly co-cultured with PC3 or ARCaP(M) cells for 24 hours prior to the collection of conditioned media, and then the co-culture conditioned media was used to treat the tumor cells (Adipo CCM; Figure 3C), a significant upregulation of glycolysis-associated genes was observed in both tumor lines (Figure 3D). This altered gene expression was mirrored by the increased levels of glycolysis-associated proteins (Figure 3E), suggesting that paracrine signaling is required between the adipocytes and tumor cells for the subsequent metabolic shift towards the glycolytic phenotype. Interestingly, inactivation of proteins in the co-culture media by boiling did not reduce the expression of glycolytic genes (Supplementary Figure 7), suggesting that the observed Warburg phenomenon might be driven by lipid rather than protein mediators.

### Functional evidence of enhanced glycolytic phenotype in response to marrow adipocytes

**Figure 4:**
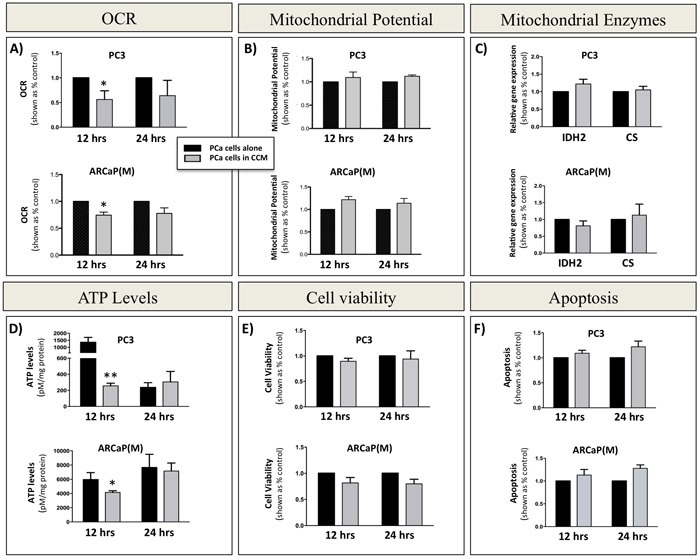
Decreased oxidative phosphorylation in prostate cancer cells exposed to bone marrow adipocyte-derived factors **A.** Seahorse XF^e24^ analyzer (Seahorse Bioscience) analysis of the oxygen consumption rate (OCR) in PC3 (top) and ARCaP(M) (bottom) cells upon 12- and 24-hour incubation in the absence or presence of CCM. **B.** Mitochondrial membrane potential measured *via* JC-1 fluorescence. **C.** Taqman RT-PCR analysis of oxidative phosphorylation genes *isocitrate dehydrogenase 2 (IDH2)* and *citrate synthase (CS)* after 12 and 24 hours in culture in the absence or presence of CCM. Data are normalized to *HPRT1* and shown relative to control. **D.** ATP levels in PC3 (top) and ARCaP(M) (bottom) cells cultured in the absence or presence of CCM. Significant decrease in ATP levels was observed after 12 hours. CCM exposure had no effect on viability as shown by Calcein AM assay **E.** and JC-1 apoptosis analyses **F.** Values *P < 0.05; **P < 0.01 are considered statistically significant.

The increased expression of glycolytic genes and proteins in tumor cells exposed to adipocytes, and significantly elevated levels of lactate in transwell co-cultures, clearly indicated augmented glycolytic activity in tumor cells interacting with adipocytes. An enhanced glycolytic phenotype in cells undergoing Warburg metabolism can often be associated with dysfunction in mitochondrial activity and consequently reduced rates of oxidative phosphorylation (OXPHOS) [56]. To determine if this is true in our system we performed an XF^e^ Seahorse analysis in tumor cells grown in the absence or presence of Adipo CCM and used oxygen consumption rate (OCR) as a tool to quantify OXPHOS. Significantly reduced OCR was detected in both PC3 and ARCaP(M) cells exposed to Adipo CCM for 12 hours. (Figure 4A). A decrease in OCR was also observable at 24 hours and did not appear to be due to a reduction in mitochondrial integrity, since there were no significant changes in JC-1 fluorescence, indicating that membrane matrices remained intact (Figure 4B). This was further supported by the lack of significant changes in expression of two mitochondrial enzymes, *CS* and *IDH2* in PCa cells exposed to Adipo CCM (Figure 4C), a result mirroring their unaltered expression *in vivo* (Supplementary Figure 3).

Since oxidative phosphorylation is much more efficient at producing copious amounts of ATP than glycolysis, a decrease in OXPHOS activity should expectedly result in a depletion of cellular ATP levels. Indeed, exposure of PC3 and ARCaP(M) cells to Adipo CCM for 12 hours led to a significant decrease in ATP concentration; however, further exposure to Adipo CCM for up to 24 hours led to a rescue of cellular ATP further suggesting an enhanced glycolytic phenotype upon Adipo CCM treatment (Figure 4D). This was further confirmed by additional recovery of ATP levels with 48-hour exposure to Adipo CCM (Supplementary Figure 8). It is important to note that the reduction in cellular ATP levels at 12 hours was not due to an enhanced proliferation induced by Adipo CCM as the Calcein AM assay showed no significant differences in cell numbers or viability of Adipo CCM-treated cells compared to cells grown under control conditions (Figure 4E). The uncompromised viability of Adipo CCM-treated tumor cells at 12 or 24 hours was further confirmed by the apoptosis assay showing no differences between control and Adipo CCM-treated cells (Figure 4F).

### Prostate cancer cells stimulate lipolysis in adipocytes

**Figure 5:**
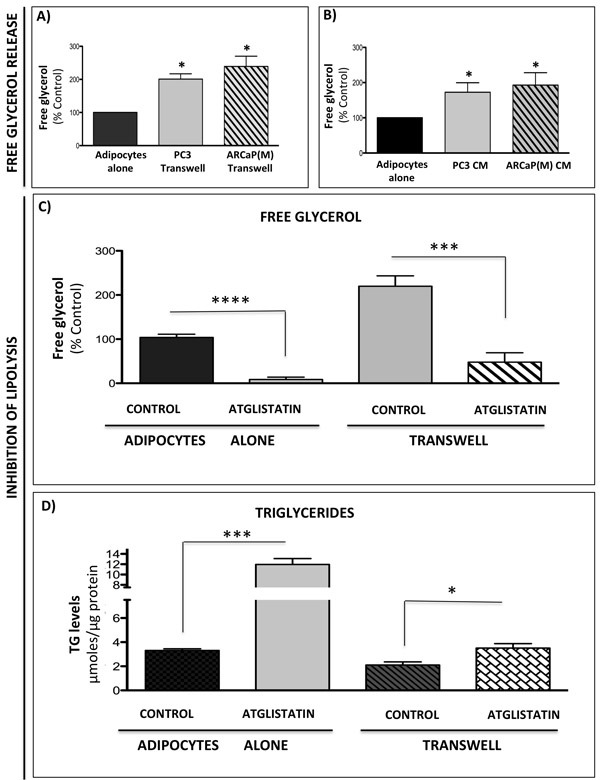
Prostate cancer cells stimulate lipolysis in bone marrow adipocytes Free glycerol release from adipocytes in transwell co-culture with PC3 or ARCaP(M) cells **A.** or adipocytes treated with conditioned media from PC3 and ARCaP(M) cells **B.** **C.** Free glycerol release by adipocytes cultured alone or in transwell with tumor cells in the absence or presence of 10μM Atglistatin. Samples were measured in triplicate and are representative of three separate experiments (shown as percent control). Data are shown as the mean ± SD. **D.** Intracellular triglyceride (TG) levels were measured in adipocytes cultured alone or in transwell with PC3 cells. Measurements were done in triplicate and are representative of three separate experiments. Data are shown as μmoles TG/μg protein in cell lysates (Mean ± SD). Values *P < 0.05; *** *P* < 0.001, and ****P < 0.0001, are considered statistically significant.

Adipocytes store triglycerides and hydrolyze them into glycerol and free fatty acids *via* the process of lipolysis [37] and lipolysis-generated glycerol can feed into the glycolytic pathway [39-41]. Based on the above-presented evidence that bone marrow adipocytes induce metabolic changes in tumor cells, and the fact that these changes appear to require paracrine interaction between the two cell types, we sought to investigate whether this could be due to tumor cell-induced lipolysis in adipocytes, as previously demonstrated in ovarian cancer [38]. We have shown previously that exposure to adipocyte-derived factors leads to lipid accumulation by prostate tumor cells [29] and lipids have been shown to contribute to the Warburg phenotype in tumor cells [32-36]. Indeed, our analysis of media from marrow adipocytes grown alone or in a transwell co-culture with tumor cells revealed significant increases in free glycerol levels under co-culture conditions (Figure 5A). Similar changes were observed when adipocytes were treated with media conditioned by PC3 or ARCaP(M) cells (Figure 5B). The master regulator and the rate-limiting enzyme driving lipolysis in adipocytes is adipose triglyceride lipase (ATGL) [57, 58]. Our analysis of gene expression of *ATGL* in adipocytes co-cultured with tumor cells or exposed to tumor cell-conditioned media showed significant upregulation indicating an induction of a lipolytic phenotype (Supplementary Figure 9) and suggesting that tumor cells may be secreting factors that induce lipolysis in fat cells. We next utilized a selective ATGL inhibitor Atglistatin, known to effectively block lipolysis in adipocytes [59] and recently shown to attenuate the growth of cancer cells [60]. A complete abrogation of free glycerol release by 10 μM Atglistatin (Figure 5C), mirrored by an accumulation of un-hydrolyzed triglycerides (Figure 5D) was observed for fat cells cultured in the absence of tumor cells. A very effective (∼80%), but not absolute, reduction in free glycerol levels was also observed in transwell co-cultures (Figure 5C). This incomplete inhibition of lipolysis in adipocytes grown in transwell co-cultures was reflected in overall lower triglyceride levels as compared to adipocytes grown alone (Figure 5D). This suggests a dynamic, paracrine interaction between the two cell types that results in ongoing hydrolysis, uptake and release of lipids.

### Inhibition of lipolysis in adipocytes is not sufficient to reverse WARBURG phenotype in tumor cells

**Figure 6:**
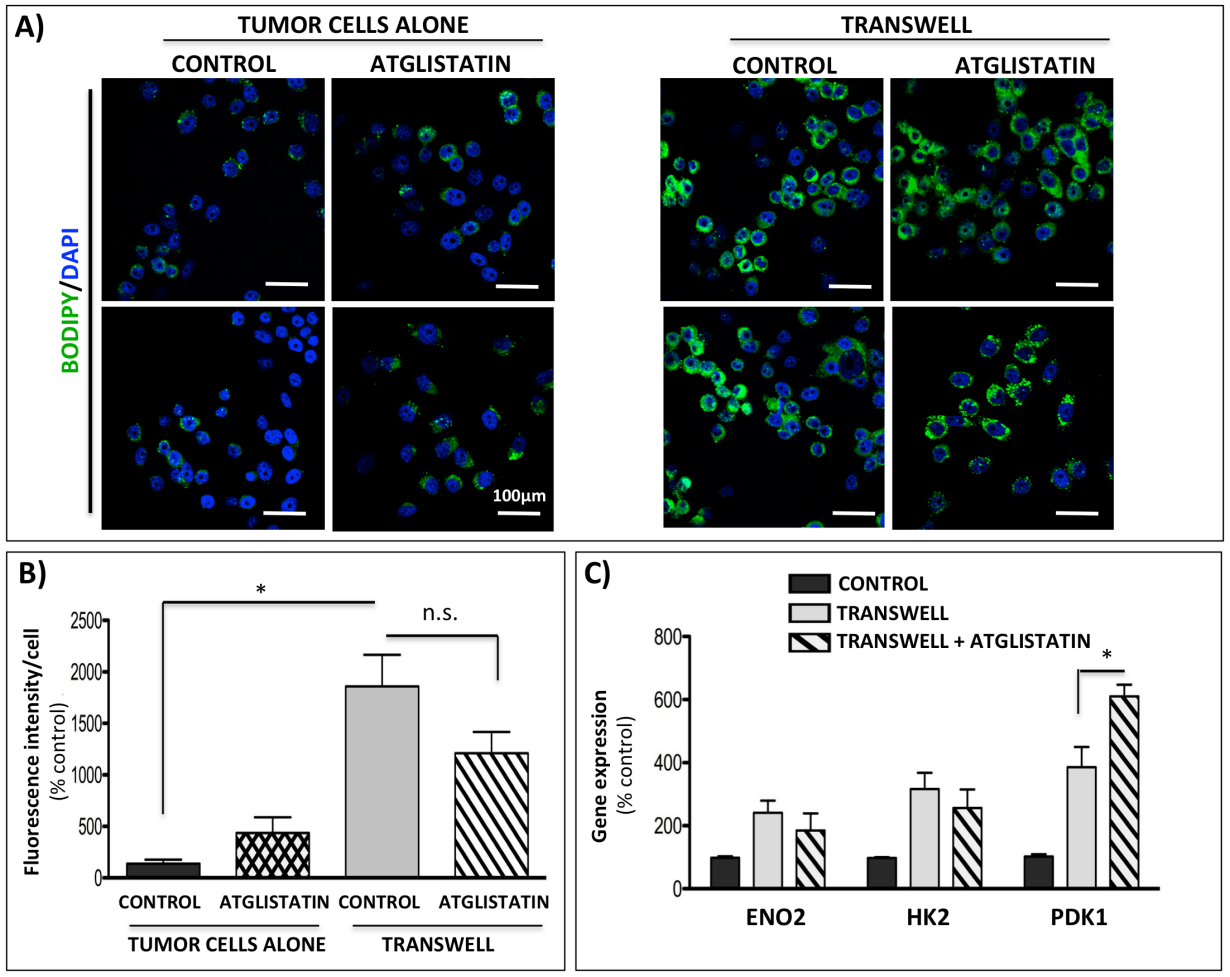
Atglistatin does not prevent lipid accumulation by the tumor cells and is not sufficient to prevent the induction of Warburg phenotype **A.** Immunofluorescence imaging of lipid droplets (BODIPY 493/503 nm) in PC3 cells alone (left panels) or in transwell co-culture with bone marrow adipocytes (right panels) and in the presence or absence of 10μM Atglistatin. DAPI was used as a nuclear stain; 40x images. Bar 100μm. **B.** Fluorescent intensity of the BODIPY 493/503 staining was quantified using Volocity (Perkin Elmer, Waltham, MA) and shown relative to PC3 cells alone. Results represent a mean of at least 3 independent experiments ± SD. **C.** Taqman RT-PCR analysis of *ENO2, HK2*, and *PDK1* in PC3 cells in transwell co-culture in the absence or presence of 10μM Atglistatin. Data are normalized to *HPRT1* and shown relative to control. Values *P < 0.05 are considered statistically significant.

Since lipolysis-generated glycerol can incorporate into the glycolytic pathway, we went on to determine whether inhibition of adipocyte ATGL with Atglistatin could reverse the Warburg phenotype in tumor cells. Our previous studies have shown that prostate tumor cells are capable of taking up adipocyte-supplied lipids [29]. To determine if this uptake can be reduced by inhibitors of lipolysis, we treated the tumor cells grown alone or in transwell co-culture with Atglistatin and performed BODIPY staining (Figure 6A). In agreement with our previous results [29], significantly increased lipid labeling was observed in tumor cells exposed to adipocytes in transwell co-culture (Figure 6A, right panels). Interestingly, treatment with Atglistatin had little effect on adipocyte-induced lipid uptake, as demonstrated by sustained BODIPY fluorescence (Figure 6A and 6B). This was further confirmed by significantly increased gene expression of lipid droplet marker *perilipin 2* and lipid transporter *CD36* in tumor cells exposed to adipocytes both in the absence and presence of Atglistatin (Supplementary Figure 10A).

Consistent with limited effects of adipocyte ATGL inhibition on lipid uptake by the tumor cells, only modest reduction in mRNA levels of *ENO2* and *HK2* was revealed indicating limited impact on glycolytic phenotype in the tumor cells. Even more surprisingly, the presence of Atglistatin in transwell co-cultures led to a small, but significant increase in the expression of *PDK1* (Figure 6C). This upregulation at the gene level corresponded to sustained higher levels of p-PDH at the protein level, suggestive of enhanced PDK1 activity in tumor cells interacting with adipocytes (Supplementary Figure 11). Interestingly, in addition to the effects on PDK1, Atglistatin treatment increased the expression of lipid transporter fatty acid binding protein 4 (FABP4) in tumor cells grown in transwell with adipocytes (Supplementary Figure 11). We have shown previously that FABP4 levels are significantly induced in tumor cells exposed to adipocyte-derived factors [29]. Given the known role of FABP4 in lipid transport and hydrolysis [61], its apparent induction by the inhibitors of lipolysis suggests a potential feedback response by tumor cells overwhelmed with adipocyte-supplied lipids. It is noteworthy that the expression of tumor-derived *monoacyl glycerol lipase (MAGL)*, a lipase previously implicated in prostate cancer progression [62, 63], was also induced in response to adipocytes and persisted upon inhibition of adipocyte ATGL with Atglistatin, suggesting an additional possible compensatory mechanism in tumor cells that might be contributing to the adipocyte-driven metabolic phenotype (Supplementary Figure 10A).

### Marrow adipocytes induce HIF-1α signaling in prostate tumor cells

**Figure 7:**
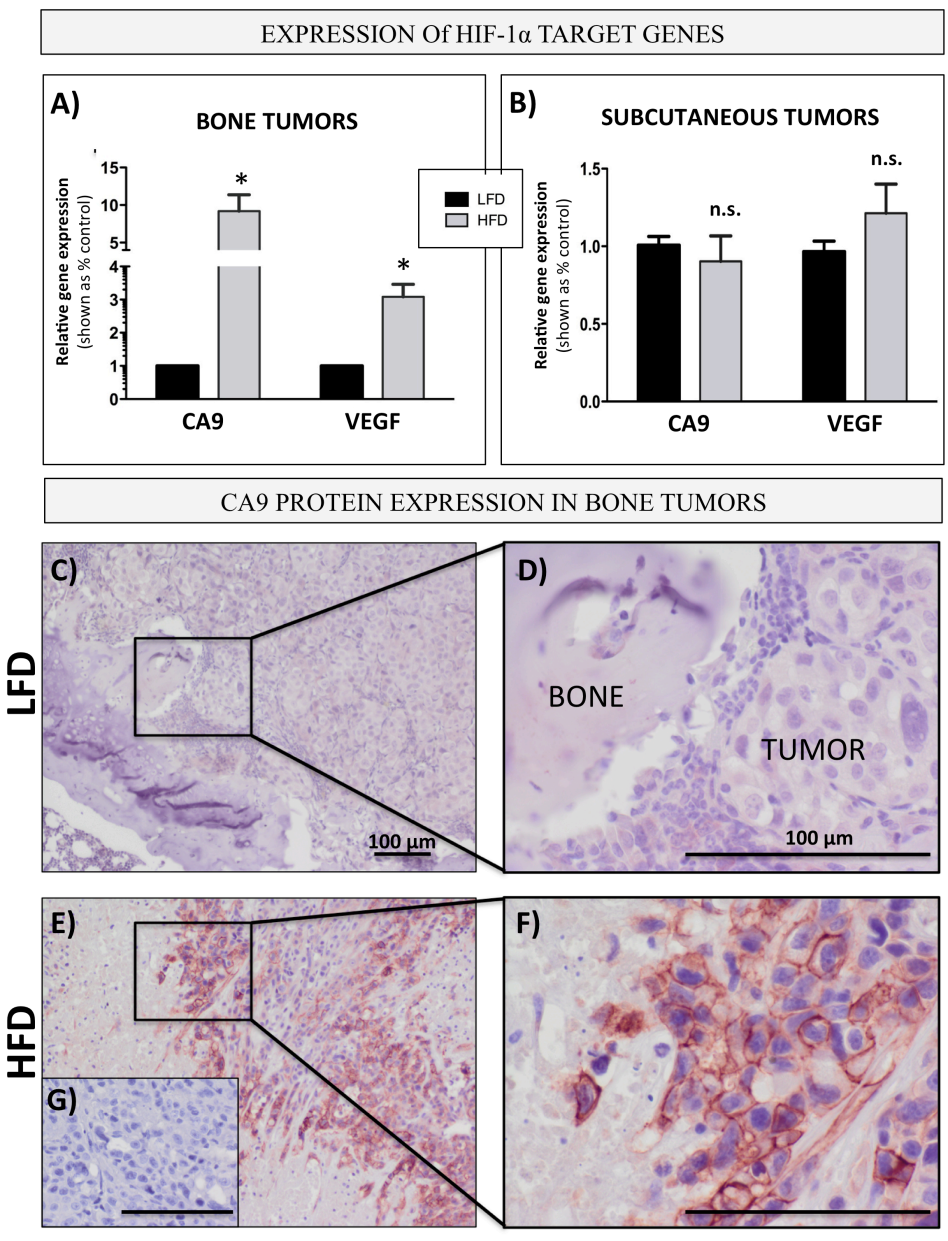
Bone marrow adiposity enhances HIF-1α signaling in PCa cells *in vivo* prostate bone tumors Taqman RT-PCR analysis of *CA9* and *VEGF* in PC3 cells grown intratibially **A.** and subcutaneously **B.** in LFD and HFD fed mice. Data are normalized to *EPCAM* and shown relative to LFD tumors. **C.**-**G.** Immunohistochemical (NovaRED) staining for CA9 protein in prostate bone tumors from mice on LFD **C.** and HFD **E.**, 10x images **D.**,**F.** High magnification (40x) images depicting membrane CA9 localization in HFD **F.** but not LFD **D.** tumors. **G.** No primary antibody control. Bar, 100μm.

One of the major mechanisms behind metabolic re-programming towards a glycolytic phenotype is the activation of HIF-1α signaling [44]. Hypoxia has been linked with aggressiveness and metastatic progression in prostate cancer [64] and we have shown previously that *HIF-1α* gene expression is increased in prostate bone tumors from HFD mice as compared to LFD mice [29]. To determine whether bone marrow adiposity might be contributing to HIF-1α activation in the bone microenvironment, we analyzed the mRNA levels of HIF-1α target genes, *CA9* and *VEGF,* in intratibial PC3 tumors from LFD- or HFD-fed mice. Both target genes were significantly upregulated in bone tumors from mice on HFD as compared to LFD mice (Figure 7A), a result that complemented a significant increase in the levels of *GLUT1* (Figure 1B), another direct target of HIF-1α activity [65]. Notably, no difference in *CA9* and *VEGF* expression between LFD and HFD conditions was observed in subcutaneous tumors (Figure 7B), in line with our earlier finding demonstrating that an increase in *GLUT1* and augmented levels of other glycolysis-associated genes are observable only in metastatic tumors but not in primary tumors in PCa patients (Figure 1A), and in bone tumors but not in subcutaneous tumors in mice (Figure 1B and 1C). We next performed an immunohistochemical analysis of CA9 expression (Figure 7C-7G). Our results showed weak, diffuse CA9 staining in bone tumors from LFD mice (Figure 7C, 7D), whereas an abundance of CA9 protein with its typical membrane localization was detected in tumors from HFD mice (Figure 7E, 7F), a result further confirming a glycolytic phenotype of bone tumors under conditions of high marrow adiposity.

**Figure 8:**
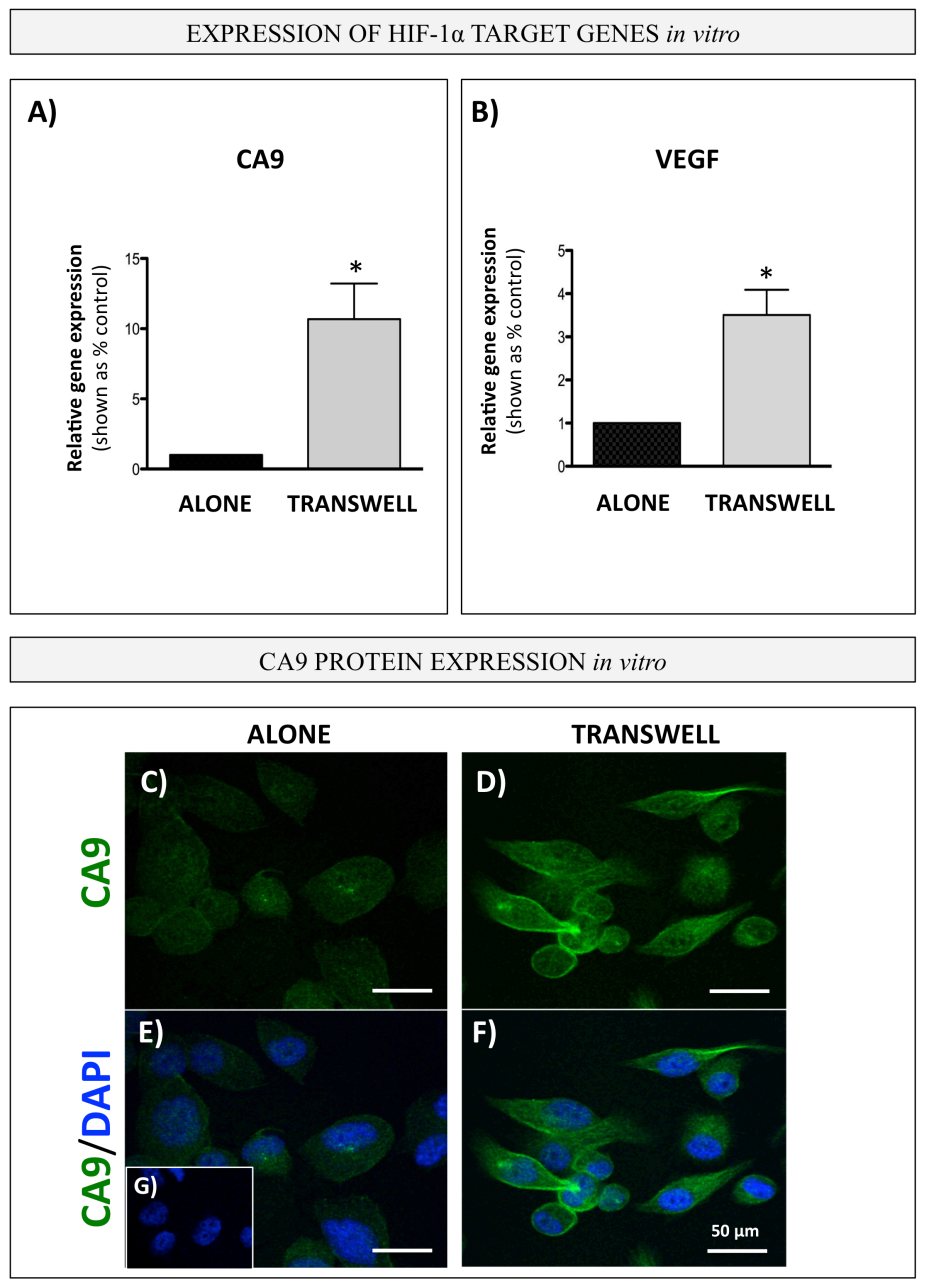
Bone marrow adipocytes activate HIF-1α signaling in PCa cells *in vitro* *Carbonic anhydrase 9* (*CA9*; **A.** and *VEGF* **B.** gene expression (Taqman RT PCR) in PC3 cells in transwell co-culture with bone marrow adipocytes. Data are representative of at least 3 separate experiments, normalized to *HPRT1* and shown relative to tumor cells cultured alone. Values *P < 0.05 are considered statistically significant. **C.**-**G.** Immunofluorescence staining of CA9 (green fluorescence) in PC3 cells grown alone (left) or in transwell co-culture with bone marrow adipocytes (right); DAPI (blue) was used as nuclear dye; 63x images. Bar, 50μm. G. No primary antibody control

To determine whether bone marrow adipocytes are in fact capable of activating HIF-1α in PCa cells, we examined the expression of *CA9* and *VEGF* in PC3 cells under transwell conditions. Indeed, expression of both genes was highly increased in cells grown in transwell co-culture with adipocytes (Figure 8A, 8B). In addition, immunofluorescence analysis of CA9 protein revealed a significant increase in expression and typical membrane localization of CA9 in PC3 cells exposed to adipocytes (Figure 8C-8G). Notably, adipocyte treatment with Atglistatin had no effect on *CA9, VEGF* or *GLUT1* expression in PC3 cells (Supplementary Figure 10B), suggesting that inhibition of adipocyte lipolysis is not sufficient to reverse adipocyte-driven HIF-1α activation in tumor cells and offers a potential explanation for the persisting glycolytic phenotype.

### HIF-1α knockdown inhibits acquisition of a glycolytic phenotype in PCa cells exposed to adipocytes

**Figure 9:**
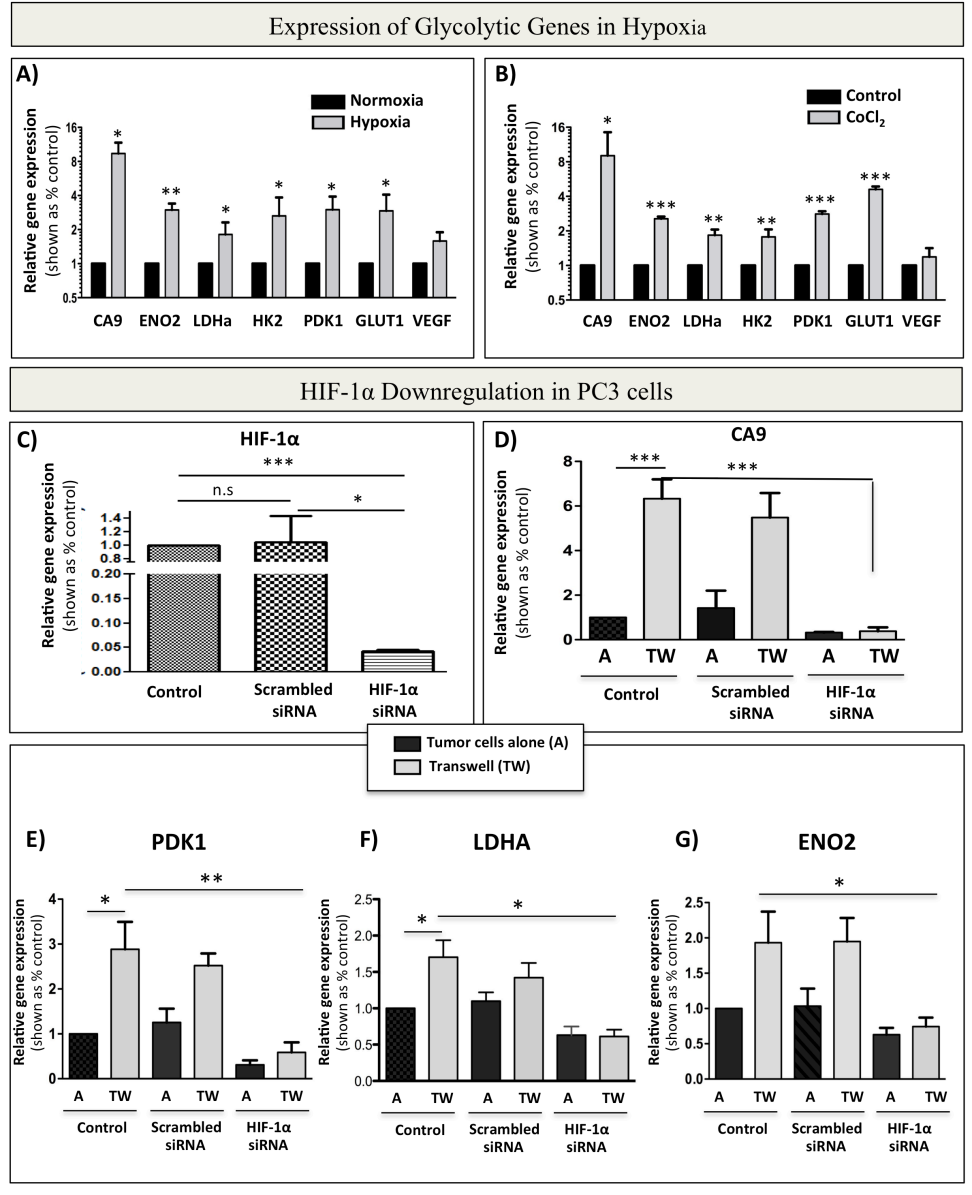
siRNA-mediated knockdown of HIF-1α abrogates bone marrow adipocyte-induced Warburg phenotype in PC3 cells **A.** Taqman RT-PCR analysis of *CA9*, *ENO2*, *LDHa*, *HK2*, *PDK1*, *GLUT1*, and *VEGF* in PC3 cells cultured in normoxia (20% O_2_) or hypoxia (1% O_2_) **B.** mRNA expression of *CA9*, *ENO2*, *LDHa*, *HK2*, *PDK1*, *GLUT1*, and *VEGF* in response to treatment with 150 μM CoCl_2._ **C.** mRNA levels of *HIF-1α* in PC3 cells grown under control conditions or treated with 20 μM scrambled siRNA, or 20 μM HIF-1α siRNA. **D.** Taqman RT-PCR analysis of the expression of HIF-1α target gene *CA9* to further confirm HIF-1α knockdown from cells grown in the presence or absence of adipocytes. E-G: Effect of HIF-1α knockdown on the mRNA expression of glycolysis associated genes: *PDK1* **E.**, *LDHA* **F.**, and *ENO2* **G.** Data are the mean of analyses with 2 different siRNA constructs done in triplicate. Values *P < 0.05; **P < 0.01, and ***P < 0.001 are considered statistically significant.

Activation and stabilization of HIF-1α is known to be a major event in metabolic transformation to a glycolytic phenotype [44]. Indeed, culture of PC3 cells under hypoxic (1% oxygen) conditions or treatment with HIF-1α inducer CoCl_2_ (Figure 9A and 9B) efficiently increase the expression of glycolytic genes to levels comparable to those observed in tumor cell-adipocyte co-cultures (Figure 2B). This suggests that adipocyte-driven Warburg phenotype in tumor cells is likely a downstream effect of HIF-1α activation under normoxic conditions. To test this, we downregulated HIF-1α in PC3 cells by siRNA and cultured control and knockdown cells alone or in transwell with marrow adipocytes (Figure 9C, 9D). A significant reduction in HIF-1α activity in siRNA-treated cells was evident by almost complete abrogation of *CA9* expression (Figure 9D). This coincided with reduced expression of glycolytic genes *PDK1, LDHA* and *ENO2* (Figure 9E-9G). Analogous to PC3 cells, ARCaP(M) cells also showed HIF-1α activation upon exposure to adipocytes, as evidenced by the increases in *CA9* mRNA expression, which is otherwise undetectable under control conditions (Supplementary Figure 12A). Exposure to adipocytes also led to augmented expression of *VEGF* (Supplementary Figure 12B), as well as increased *GLUT1* (Figure 2B). Upon siRNA-mediated knockdown of HIF-1α (Supplementary Figure 12C), expression of *CA9, PDK1, LDHA* and *ENO2* was significantly reduced (Supplementary Figure 12D-12G), further underscoring the importance of HIF-1α signaling in marrow adipocyte-driven metabolic adaptation of PCa tumors in bone.

## DISCUSSION

Adipocytes are metabolically active cells with the ability to regulate the phenotype and function of neighboring cancer cells through the processes of lipid transfer and lipolysis [6, 38, 66-68]. They have been linked to metabolic reprogramming and tumor progression in a handful of cancers, including tumors of breast, ovaries and colon, all with tendencies to grow in fat-enriched sites [6, 69-72]. In the context of prostate cancer, adipocytes from visceral and periprostatic tissues have been linked to the progression of localized disease [6, 73]. The data have been lacking, however, on how adipocytes that occupy bone marrow space might be influencing the metabolism and consequently the progression of prostate tumors that have colonized this fat-enriched metastatic niche. The results presented above reveal an important contribution of bone marrow adipocytes to the metabolic phenotype of metastatic PCa tumors. We show that marrow fat cells are capable of inducing the glycolytic phenotype in PCa cells through paracrine upregulation of glycolytic enzymes, increases in lactate secretion and reduction in oxidative phosphorylation. We also demonstrate that tumor cells are able to modulate the metabolism of a fat cell. They do so by stimulating adipocyte lipolysis in the effort of utilizing the fat cell-supplied lipids to fuel the glycolytic pathway. This speaks to the importance of the supportive host microenvironment in tumor progression and demonstrates the metabolic coupling between the tumor cells and host adipocytes. This adipocyte-tumor cell interaction ultimately shapes the metabolism of the tumor cell allowing for the adaptive survival in the metastatic niche [31].

We focused on metabolism because of the important selective advantage an enhanced glycolytic phenotype can have on tumor aggressiveness and survival within a harsh metastatic niche [74, 75]. Glycolysis is not the most effective way, but it is the quickest way of creating ATP that allows the tumor cells to efficiently gain metabolic autonomy in the tumor microenvironment [75]. It permits the continuous supply of nutrients for biosynthetic processes, protection from oxidative stress, and, potentially, an activation of survival pathways [76, 77]. Warburg metabolism is often associated with a more hypoxic tumor signature, which is also a very well-documented facilitator of tumor aggressiveness and chemoresistance [78-81]. Glycolytic enzymes, such as ENO2, LDHa, PDK1, and HK2, and proteins involved in glucose uptake, such as GLUT1, are all regulated through HIF-1α [82-84]. In hypoxia, HIF-1α compromises oxygen-consuming OXPHOS by inducing the expression of PDK1 and preventing conversion of pyruvate into acetyl-CoA [85, 86]. The resulting production and secretion of lactate by highly glycolytic cells is known to increase tumor invasion, but it can also serve as an alternative carbon source for surrounding oxygenated cells [87]. Hypoxia is also known to induce acidosis *via* increased acid load in the tumor microenvironment, a process that leads to upregulation of enzymes, such as carbonic anhydrase 9 (CA9), that can regulate extracellular pH allowing the tumor cells to thrive in the acidic microenvironment. [75]. It is the membrane-bound CA9, whose expression correlates with aggressive disease and poor survival in many cancers [75], that is thought to modulate pH through the interaction with bicarbonate transporters on the cell surface [88]. Notably in our study, evidence of clearly increased CA9 expression in human metastatic prostate cancer samples and experimental bone tumors from HFD mice, together with immunocytochemical data showing its surface expression in response to adipocytes, suggest that metastatic PCa cells might be utilizing CA9-dependent mechanisms to adapt and grow in the metastatic niche.

Bone tissue is intrinsically hypoxic [89, 90], with O_2_ concentrations ranging from ∼1.3 to 3% based on the proximity to the vessels and distance from the endosteum [91]. This makes bone an environment already prone to hypoxic stress. Our data presented herein show that PCa cells exposed to low oxygen concentration in a hypoxia chamber show the same glycolytic phenotype as cells interacting with marrow adipocytes under normoxic conditions. This suggests that adipocytes promote oxygen-independent mechanism of HIF-1α activation in PCa cells, known as ‘pseudohypoxia’ [49, 92, 93]. HIF-1α, under some circumstances, can be directly activated in well-oxygenated microenvironments [94], or its activation and stabilization can be a consequence of mutations in metabolic genes [49, 95]. The mechanisms behind its regulation by bone marrow adipocytes are currently unknown and are subject of ongoing investigations in our laboratory.

One important consequence of hypoxia is the induction of HIF-1α-mediated accumulation of lipid droplets in tumor cells [96]. Hypoxic tumor cells have been recently shown to bypass lipogenesis and to rely on scavenging of unsaturated lipids from the microenvironment [97, 98].

Hypoxia has also been linked to the upregulation of proteins that stabilize the integrity of lipid droplets, such as perilipin and adipose differentiation-related protein (ADRP), as well as members of the FABP4 family of lipid transporters [96]. This is of relevance to our study, as hypoxia and glycolytic phenotype in our PCa cells interacting with adipocytes coincide with both an enhanced lipid uptake and an upregulation of lipid transporters and lipid droplet markers. Whether HIF-1α activation is a cause or a consequence of lipid accumulation in metastatic tumor cells remains to be uncovered. It is plausible that the initial exposure to adipocyte-supplied lipids triggers HIF-1α stabilization and that consequent activation of HIF-1α signaling leads to further lipid uptake, perpetuating the hypoxic and glycolytic phenotype in tumor cells. The fact that hypoxia-mediated effects persist even upon the inhibition of adipocyte-driven lipolysis speaks to the importance of HIF-1α signaling in driving the Warburg phenotype in tumor cells. The mechanism by which tumor cells exposed to adipocytes in the presence of ATGL inhibitor continue to accumulate and utilize fat cell-supplied lipids remains to be elucidated. One potential player in this process might be the MAGL, an enzyme implicated in lipid remodeling and scavenging by tumor cells and shown recently to be associated with aggressive phenotype of PCa cells [62, 63, 99].

Although the acquisition of a glycolytic phenotype appears to be the predominant metabolic change in PCa cells in response to marrow adipocytes, it is important to mention that some of the glycolytic enzymes we see upregulated in response to fat cells are also known to have non-glycolytic functions that are important for tumor cell growth and survival. Specifically, hexokinase-2 (HK2), an enzyme critical for first step of glycolysis, elicits its functions by binding to the outer mitochondrial membrane protein voltage-dependent anion channel (VDAC). This allows for receipt of newly synthesized ATP and rapid and efficient production of glucose-6-phosphate, which contributes not only to the glycolytic pathway but also to metabolite synthesis in the pentose-phosphate pathway and TCA cycle, both important for tumor growth and proliferation [100-103]. Intriguingly, interaction of HK2 with VDAC prevents pro-apoptotic proteins such as BAX and BAD from binding to the mitochondrial pores to facilitate apoptosis resulting in cells that are more resistant to cell death and chemotherapy [104-106]. Because around 80% of total HK2 is reported to be bound to the mitochondrial VDAC [107], and because we see elevated levels of HK2 in tumor cells that are exposed to bone marrow adipocytes, it is plausible to expect that the mitochondrial binding of HK2 is occurring in PCa cells, a process that could be promoting tumor cell survival *via* inhibition of intrinsic apoptosis in response to adipocyte-supplied factors.

In this study we utilized an intratibial model of intraosseous tumor growth, a widely used *in vivo* experimental system designed to specifically study tumor-bone interactions, and tumor growth and expansion in the bone microenvironment [108-110]. We and others have used this system previously in combination with diet induced obesity (DIO) models to study effects of marrow adiposity on tumor progression in bone [29, 53, 111]. The DIO model is a well-documented approach to induce marrow adiposity [18, 29, 51, 52] and we have previously shown that eight-week exposure to HFD significantly augments adipocyte numbers in this system [29, 53]. We do acknowledge we cannot exclude potential systemic consequences of the diet itself on both the tumor growth and metabolic phenotype in bone. There is an ongoing debate on the role of dietary lipids in prostate cancer development and progression [112, 113] and future studies utilizing genetic models of obesity and age-induced models of marrow adiposity will provide a more detailed understanding of adipocyte impact on metabolic adaptation and survival of tumor cells in the bone marrow niche. An additional value will be added by the comparative metabolic profiling of experimental bone prostate tumors and orthotopic primary prostate tumors.

The rationale for our study was based on the Oncomine analyses of human prostate cancer samples suggesting clearly distinct metabolic phenotype of metastatic sites as compared to primary tumors. We recognize that currently available datasets do not allow for distinction of bone metastases from other potential metastatic sites. However, given the fact that more than 80% of metastatic patients present with bone lesions, it is highly likely that majority of these tumors represent skeletal lesions. Limited availability of bone metastatic tissues is certainly an ongoing, unresolved issue in prostate cancer research. We believe that this distinct metabolic phenotype in metastatic tissues revealed by our Oncomine analyses provides an important starting point for future studies investigating the contribution of tumor metabolism to progression and survival of metastatic prostate tumors in the bone microenvironment.

Metabolic requirements of a tumor cell are much more complex than previously appreciated and they likely involve multiple pathways and nutrients that aid in malignant transformation and progression [114]. There is also no doubt that metabolic adaptation and consequent growth and survival of a tumor is the result of a complex interaction between the cancer cell and the surrounding host microenvironment. Data presented herein reveal marrow adipocytes as important players involved in shaping tumor metabolism in bone. To our knowledge, this is the first study demonstrating the importance of bi-directional interactions between marrow fat cells and tumor cells in activating HIF-1α signaling and driving the Warburg phenotype in metastatic prostate cancer cells. Adipocyte-supplied factors have been shown to enhance glycolysis in primary cancer cells and render them more aggressive and resistant to therapy [115-118]. Understanding the molecular mechanisms behind this metabolic regulation in bone is of critical importance in terms of potential treatment options for metastatic disease.

## MATERIALS AND METHODS

### Materials

Dulbecco's modified Eagle's medium (DMEM), RPMI-1640 medium, and other chemicals, unless otherwise stated, were obtained from Sigma (St. Louis, MO). HyClone fetal bovine serum (FBS) was from ThermoFisher (Pittsburg, PA). Trypsin-EDTA, collagenase, BODIPY (493/503), Gentamicin (G418), Alexa Fluor 488-conjugated goat anti-rabbit IgG, and rabbit anti-human FABP4 were from Invitrogen (Carlsbad, CA). PureCol^®^ collagen type I was from Advanced Biomatrix (San Diego, CA). Mouse monoclonal E7 Beta tubulin antibody was from Developmental studies Hybridoma Bank (Iowa City, IA). StemXVivo Adipogenic Suppliment, was from R&D Systems (Minneapolis, MN). Rabbit anti-human/mouse Δ-actin antibodies were from Novus Biologicals (Littleton, CO). Mouse anti-human neuron-specific Enolase was from Dako-Agilent Technologies (Denmark). Rabbit anti-human-pyruvate dehydrogenase kinase, lactate dehydrogenase alpha, rabbit anti-human hexokinase 2 were from Cell Signaling Technologies (Beverly, Massachusetts). Rabbit anti-human phosphorylated pyruvate dehydrogenase, rabbit monoclonal anti-carbonic anhydrase 9 antibody, and the fluorometric L-lactate detection kit were from Abcam (Cambridge, UK). Adipolyze lipolysis detection kit was from Lonza (Switzerland). RNeasy Mini Kits were from Qiagen (Valencia, CA). Immunoblotting “Western Lightning ECL Plus” detection kits were from Perkin Elmer LLC (Waltham, MA). Rosiglitazone and the Triglyceride Colorimetric Assay kit were from Cayman Chemical (Ann Arbor, MI). ImmPACT NovaRED Peroxidase Substrate and ImmPRESS Anti-Rabbit Peroxidase Reagent kit were from Vector Laboratories (Burlington, CA). Cobalt Chloride was from MP Biomedicals (Solon, OH). Atglistatin was from Axon Medchem (Groningen, Netherlands).

### Cell lines

PC3 cell line, derived from a bone metastasis of a high-grade adenocarcinoma [119], and DU145 cells, derived from human prostate adenocarcinoma metastatic to the brain [120], were purchased from American Type Culture Collection (ATCC; Manassas, VA). The ARCaP(M), an Androgen-Repressed Metastatic Prostate Cancer Cells M (‘Mesenchymal’ Clone) [121] were purchased from Novicure Biotechnology (Birmingham, AL). The human prostate cancer C4-2B cell line was kindly provided by Dr. Leland W. K. Chung, Cedars-Sinai Medical Center (Los Angeles, CA). PC3 and DU145 cells were grown in DMEM medium with 10% FBS, ARCaP(M) cells in RPMI medium with 5% FBS, and C4-2B cells in RPMI medium with 10% FBS. Cells were maintained in a 37°C humidified incubator ventilated with 5% CO_2_.

Primary mouse bone marrow stromal cells (mBMSC) were isolated from femurs and tibiae of 6- to 8- week old FVB/N mice according to previously established protocols [122]. To induce bone marrow adipocyte differentiation, mBMSC cells were plated in 3D collagen I gels, grown to confluency for 48-72 hours and treated with adipogenic cocktail (30% StemXVivo Adipogenic Suppliment, 1 μM insulin, 2 μM Rosiglitazone; DMEM and 10% FBS) for 8-10 days as previously described [29]. Differentiated bone marrow adipocyte cultures were washed 3 times with PBS and used in experiments.

### Animals

All experiments involving mice were performed in accordance with the protocol approved by the institutional Animal Investigation Committee of Wayne State University and NIH guidelines. *In vivo* xenograft studies were performed in male mice in the FVB/N background with homozygous null mutation in the Rag1 gene [FVB/N/N5, Rag-1^−/−^]. All mice were bred in-house.

### Diets

At 5 weeks of age, mice caged in groups of 4 were started on either a low-fat (LFD) diet (10% calories from fat; Research Diets no. D12450Ji) or a high-fat (HFD) diet (60% calories from fat; Research Diets no. D12492i). D12450Ji is a standard matched control diet for D12492i as recommended by Research Diets. Mice were maintained on respective diets for 8 weeks prior to the tumor implantation and continued on the diets for additional 6 weeks (PC3 tumors) or 8 weeks (ARCaP(M) tumors). Where indicated, mice were switched from HFD to LFD at time of tumor implantation and maintained on LFD for the remainder of the experiment.

### Intratibial and subcutaneous injections of prostate cancer cells

Intratibial tumor injections were performed under isoflurane inhalational anesthesia according to the previously published procedures [29, 53, 123]. Briefly, a cell preparation containing 5 ×10^5^ of PC3 /ARCaP(M) cells in PBS (20 μl, right tibia), or PBS alone (control, 20 μl, left tibia) was injected into the bone marrow. Mice were euthanized six weeks (PC3 cells) or eight weeks (ARCaP(M) cells) post-injection, and control and tumor-bearing tibiae were removed. For microenvironmental control, separate groups of LFD and HFD mice were injected subcutaneously with 50 μl of PC3 cell suspension (5 x10^5^ cells in PBS/Cultrex). Half of the intratibial tumor samples from each group and half of each subcutaneous tumor were fixed in Z-fix, bone tumors were decalcified, and all samples were embedded in paraffin. Remaining tissues were snap-frozen in liquid nitrogen, powderized using a tissue pulverizer and RNA was isolated using Trizol and RNeasy Mini Kit.

### TaqMan RT-PCR analyses

The cDNA from cells and *in vivo* samples was prepared from 1-2 μg of total RNA using High-Capacity cDNA Reverse Transcription kit (Applied Biosystems). The analyses of genes associated with glycolysis, lipolysis, hypoxia and mitochondrial markers were performed using TaqMan^®^ Individual Gene Expression assays for Human *ENO2* (Hs00157360), *LDHa* (Hs00855322), *HK2* (Hs00606086­), *PDK1* (Hs01561850), *GLUT1* (Hs00892681), *CS* (Hs 02574374), *IDH2* (Hs00158033), *HIF-1α* (Hs00153153), *CA9* (Hs00154208), *MAGL* (Hs00200752), *CD36* (Hs01567185), *Perillipin 2* (Hs00605340), and *VEGF* (Hs00900055). Assays were done on three biological replicates using TaqMan^®^ Fast Universal PCR Master Mix and 50 ng of cDNA/well and all reactions were run on an Applied Biosystems StepOnePlus™ system. Three biological replicates of each sample were pooled together and assays were run in at least triplicate. The same assays (*ENO2, LDHa, PDK1, HK2, GLUT1, CA9* and *VEGF*) were performed on triplicate samples of PC3 bone tumors from LFD and HFD mice and normalized to human epithelial cell marker *CD326* (*EPCAM*) (Hs00901885). Specificity of each Taqman probe was cross-checked against RNA from control mouse bones and murine adipocytes (Supplementary Table 4). For all human genes *in vitro*, data were normalized to hypoxanthine phosphoribosyltransferase (*HPRT1*; Hs02800695). For assessment of adipocyte-specific genes in adipocytes grown in co-culture with tumor cells, the following murine Taqman assays were used: *HSL* (Mm00495359) and *ATGL* (Mm00503040). Data were normalized to *Adiponectin* (Mm00456425). DataAssist™ Software (Applied Biosystems) was used for all analyses. *CA9* and *CD326* primers (IDT, Coralville, IA) for PCR were used according to manufacturer's protocol. Human *CA9* forward and reverse primer sequences are as follows: Forward: 5′-GGGTGTCATCTGGACTGTGTT-3′; Reverse: 5′-CTTCTGTGCTGCCTTCTCATC-3′. *CD326* forward and reverse primers are as follows: Forward: 5′-CTG GCC GTA AAC TGC TTT GT-3′; Reverse: 5′-AGC CCA TCA ATT GTT CTG GAG-3′.

### *In vitro* models

#### Transwell co-culture

The mBMSC cells were embedded in Collagen, plated in 6-well plates, differentiated into adipocytes, and tumor cells were seeded on top of a Transwell filter (0.2 μm pore size) to allow sharing of soluble factors between the two cell types. After 48 hours, tumor cells were washed with PBS, trypsinized and harvested for RNA and protein extraction. Adipocytes were collected using 1% collagenase. For protein analyses, lysates were re-suspended in SME buffer with protease and phosphatase inhibitors [29, 53]. For RT PCR analyses, cells were collected into RLT buffer and RNA was purified using RNeasy Mini Kit [29, 53].

#### Direct co-culture

Adipocytes embedded in Collagen I were differentiated in 100 mm dishes as previously described [29]. 600,000 PC3 or ARCaP(M) cells were plated in co-culture with adipocytes and on top of Collagen I without adipocytes as control. After 48 hours, 1% collagenase was used to break down the Collagen I and isolate the cells. Human specific qPCR probes were used to measure transcriptional responses in glycolytic genes.

#### Co-culture CM treatment

Conditioned media was obtained from either adipocytes alone (Adipo CM) or from PCa-adipocyte direct co-cultures (CCM) and either stored in −80 °C or used fresh after collection. PCa cells were seeded at 200,000 cells per well in 6-well plates 24 hours prior to treatment, then treated with either fresh DMEM containing 10% FBS Adipo CM, or CCM. After 24 hours of treatment, the cells were washed with PBS and collected for RNA and protein as previously described.

### Immunoblot analyses

Lysate and media samples were loaded based on DNA/protein concentrations and the corresponding lysates were electrophoresed on 12% or 15% SDS-PAGE gels, transferred to PVDF membranes and immunoblotted for ENO2 (1:1,000), LDHa (1:1000), PDK1 (1:500), p-PDH (1:1000), HK2 (1:1,000), FABP4 (1:500), Tubulin (1:1000), and β-actin (1:1,000). All horseradish peroxidase-labeled secondary antibodies were used at 1:10,000. Quantification and analyses of bands were performed using a Luminescent Image Analyzer LAS-1000 Plus (Fujifilm, Stamford, CT).

### CA9 immunohistochemical analyses

Tumor-bearing tibiae from LFD and HFD mice were fixed, decalcified, and embedded in paraffin. Deparaffinized and rehydrated tissues were then analyzed by immunohistochemistry for expression and localization of CA9 (rabbit anti-human CA9; 1:250). ImmPRESS Anti-Rabbit Peroxidase Polymer Detection systems along with a NovaRED kit as a substrate were used for the peroxidase-mediated reaction.

### Immunofluorescence analyses

Cells were plated on coverslips (50,000 per coverslip) in a 24-well plate, allowed to attach for at least 4 hours, and transferred to control or transwell wells. After 48 hours, cell were stained with BODIPY (493/503) by washing with PBS, fixing with 3.7% formaldehyde at RT for 40 minutes, and incubating with BODIPY (493/503) (1:1000) at RT for 1 hour. Coverslips were washed and mounted onto slides using Vectashield with DAPI (Vector Laboratories). Images were taken using a Zeiss LSM 510 META NLO confocal microscope (Carl Zeiss AG, Göttingen, Germany) and a 40 × oil immersion lens. For CA9 staining, cells were washed with PBS and fixed with cold methanol. Coverslips were stained with rabbit monoclonal anti-CA9 antibody (1:50) at 4°C overnight. Alexa Fluor 488-conjugated goat anti-rabbit IgG (1:1,000) was used as a secondary antibody, and DAPI was used as a nuclear stain. Coverslips were mounted using Vectashield and imaged using a Zeiss LSM 510 META NLO confocal microscope using a 63 × oil immersion lens.

### ATP analysis

Cells were seeded in 6-well dishes, cultured overnight and treated with either fresh media (control conditions) or CCM. At 12 and 24 hours, the cells were washed and scraped into PBS. The cells were collected in a timely manner to ensure reliability of the sample, snap frozen in liquid nitrogen and stored in −80 °C. The ATP Bioluminescence Assay Kit HS II (Roche Applied Science) and the boiling method for ATP release were used [124]. Briefly, 700 μl of incubation buffer was added to the cells (100 mM Tris-Cl, pH 7.75, 4 mM EDTA) and the solution was immediately transferred to a boiling water bath for 2 minutes. Samples were diluted 1:25 and 40 μl aliquots were used to determine the ATP concentration following the manufacturer's protocol. The experiments were done as biological duplicates, and then two aliquots were taken from each sample and assayed in triplicate. The concentrations were normalized to total protein using NanoDrop 2000 (Thermofisher Scientific). Data are shown as mean ± SD.

### Assessment of lactate levels in media

Conditioned media was obtained from PC3 and ARCaP(M) cells alone and in transwell with adipocytes after 48-hour co-culture. The media was heat-inactivated at 65 °C for 8 minutes. Abcam's L-lactate Detection Kit was used and conditioned media was assayed using a TECAN plate reader (535nm/590nm) according to manufacturer's instructions. Data were normalized to the total DNA or RNA concentrations in cell lysates. Experiments were done in triplicate and reported as mean ± SD.

### Seahorse analysis

PC3 and ARCaP(M) cells were plated on a Collagen I matrix at concentrations of 30,000 cells per well in XF^24^ Cell Microplates and cultured overnight. The following day, cells were treated with fresh DMEM medium or Co-culture CM (CCM) for 12 and 24 hours. One hour prior to reading the plate, the media was changed to DMEM containing 10 mM glucose and 2 mM glutamine. Basal readings were performed on the Seahorse analyzer and the third reading of each experiment was used. Experiments were done in triplicate with at least three wells per sample condition and reported as mean ± SD.

### Determination of mitochondrial membrane potential/ intrinsic apoptosis

The JC-1 probe (Thermofisher Scientific) was used to assess mitochondrial membrane potential as a measure mitochondrial integrity [125] Cells were plated in 96-well black plates at a density of 5,000 cells/well and grown overnight. The following day, media was replaced with either fresh DMEM or CCM and cells were allowed to incubate for 12 and 24 hours. JC-1 probe was then added at a final concentration of 1 μM to the media and the plates were incubated for 20 minutes. The plates were then read at excitation and emission wavelengths of 535 nm and 595 nm, respectively, for the red fluorescence and excitation and emission wavelengths of 485 nm and 535 nm, respectively, for the green fluorescence. Data were normalized based on cell viability. Experiments were done in triplicate with quadruplicate wells per condition at each time point and shown as mean ± SD.

### Determination of cell viability

Calcein AM Assay (Trevigen) was used to assess cell viability. Cells were seeded in black-walled 96-well plates at a density of 5,000 cells/well and grown overnight. The following day, media was removed and fresh DMEM or CCM was added to the wells and the cells were incubated for 12 or 24 hours. The plate was then read at excitation and emission wavelengths of 490 nm and 520 nm, respectively. Experiments were done in at least triplicate with quadruplicate wells analyzed per experiment and shown as mean ± SD.

### Free glycerol assay analysis

Conditioned media was obtained from adipocytes alone, adipocytes in transwell with PC3 or ARCaP(M) cells, or adipocytes treated with conditioned media from PC3 or ARCaP tumor cells and analyzed using manufacturer's protocol for the AdipoLyze Lipolysis Detection Kit (Lonza). Experiments were done in triplicate and reposted as mean ± SD.

### Triglyceride assay

Tumor cells were grown in transwell with adipocytes in the presence or absence of 10 μM Atglistatin. After 48 hours, adipocytes were collected as previously indicated and re-suspended in the Standard Diluent (provided in the Triglyceride assay kit; Cayman Chemical). Samples were sonicated and centrifuged. The supernatant was then used for the assay. All steps were performed according to manufacturer's protocol. Experiments were done in triplicate and reported as mean ± SD.

### Activation of HIF-1α *in vitro*

PC3 cells were pre-plated in 6-well plates and allowed to settle overnight. For pharmacological HIF-1α activation, cells were treated the following day with 150 μM cobalt chloride (CoCl_2_). After 24 hours, cells were lysed, and processed for RNA analyses as described above. For establishment of hypoxic cultures, cells were plated in 6-well plates and allowed to settle overnight in normoxia, and then either maintained in normoxia (control cells) or grown in Biospherix hypoxia chamber (Biospherix, Parish, NY) under. 1% O_2_ (hypoxic cells). After 24 hours, all cells were processed for RNA isolation as previously described.

### siRNA Approaches

PC3 or ARCaP(M) cells were pre-plated in 6-well plates or on Transwell filters and grown overnight. The following day, when the cells reached ∼70% confluency, a unique 27mer siRNA duplex targeting HIF-1α transcripts (OriGene-SR302102) or Trilencer-27 Universal scrambled negative control (Origene-SR30004) were added using RNAiMAX transfection reagent (Thermofisher Scientific) at a final concentration of 20 μM (based on manufacturer's protocol). After 6 hours, cells were washed and moved into transwell co-culture with differentiated bone marrow adipocytes or grown alone. After 24 hours, cells were collected and processed for RNA analyses as described above. Two unique 27mer siRNA duplexes that efficiently knocked down HIF-1α transcripts were used.

### *In silico* analyses

The Oncomine database (Oncomine^TM^ v4.5: 729 datasets, 91,866 samples) was used for the analysis of primary (P) *vs*. metastatic (M) tumors by employing filters for selection of conditions and genes of interest (prostate cancer; metastasis *vs*. primary; genes). Data were ordered by ‘overexpression’ and the threshold was adjusted to *P*-value < 1E^−^4; fold change, 2 and gene rank, top 10%. For each database, only genes that met the criteria for significance were reported.

### Statistical analyses

Data were presented as means ± SD and statistically analyzed using unpaired student *T*-test. For three or more groups, one-way analysis of variance was used.

## SUPPLEMENTARY MATERIAL TABLES AND FIGURES



## References

[1] Cairns RA, Harris IS, Mak TW (2011). Regulation of cancer cell metabolism. Nature reviews.

[2] Dakubo GD (2010). The Warburg Phenomenon and Other Metabolic Alterations of Cancer Cells. Mitochondrial Genetics and Cancer.

[3] Warburg O (1956). On the origin of cancer cells. Science (New York, NY).

[4] Levine AJ, Puzio-Kuter AM (2010). The control of the metabolic switch in cancers by oncogenes and tumor suppressor genes. Science.

[5] Hanahan D, Weinberg RA (2011). Hallmarks of cancer: the next generation. Cell.

[6] Nieman KM, Romero IL, Van Houten B, Lengyel E (2013). Adipose tissue and adipocytes support tumorigenesis and metastasis. Biochim Biophys Acta.

[7] Zadra G, Photopoulos C, Loda M (2013). The fat side of prostate cancer. Biochim Biophys Acta.

[8] Liu Y (2006). Fatty acid oxidation is a dominant bioenergetic pathway in prostate cancer. Prostate cancer and prostatic diseases.

[9] Luo J, Zha S, Gage WR, Dunn TA, Hicks JL, Bennett CJ, Ewing CM, Platz EA, Ferdinandusse S, Wanders RJ, Trent JM, Isaacs WB, De Marzo AM (2002). Alpha-methylacyl-CoA racemase: a new molecular marker for prostate cancer. Cancer Res.

[10] Zha S, Ferdinandusse S, Hicks JL, Denis S, Dunn TA, Wanders RJ, Luo J, De Marzo AM, Isaacs WB (2005). Peroxisomal branched chain fatty acid beta-oxidation pathway is upregulated in prostate cancer. Prostate.

[11] Costello LC, Liu Y, Franklin RB, Kennedy MC (1997). Zinc inhibition of mitochondrial aconitase and its importance in citrate metabolism of prostate epithelial cells. J Biol Chem.

[12] Menendez JA, Lupu R (2007). Fatty acid synthase and the lipogenic phenotype in cancer pathogenesis. Nature reviews.

[13] Rossi S, Graner E, Febbo P, Weinstein L, Bhattacharya N, Onody T, Bubley G, Balk S, Loda M (2003). Fatty acid synthase expression defines distinct molecular signatures in prostate cancer. Mol Cancer Res.

[14] Oyama N, Akino H, Suzuki Y, Kanamaru H, Sadato N, Yonekura Y, Okada K (1999). The increased accumulation of [18F]fluorodeoxyglucose in untreated prostate cancer. Japanese journal of clinical oncology.

[15] Pertega-Gomes N, Felisbino S, Massie CE, Vizcaino JR, Coelho R, Sandi C, Simoes-Sousa S, Jurmeister S, Ramos-Montoya A, Asim M, Tran M, Oliveira E, Lobo da Cunha A (2015). A glycolytic phenotype is associated with prostate cancer progression and aggressiveness: a role for monocarboxylate transporters as metabolic targets for therapy. J Pathol.

[16] Hardaway AL, Herroon MK, Rajagurubandara E, Podgorski I (2014). Bone marrow fat: linking adipocyte-induced inflammation with skeletal metastases. Cancer Metastasis Rev.

[17] Lecka-Czernik B (2011). Marrow fat metabolism is linked to the systemic energy metabolism. Bone.

[18] Rosen CJ, Ackert-Bicknell C, Rodriguez JP, Pino AM (2009). Marrow fat and the bone microenvironment: developmental, functional, and pathological implications. Crit Rev Eukaryot Gene Expr.

[19] Justesen J, Stenderup K, Ebbesen EN, Mosekilde L, Steiniche T, Kassem M (2001). Adipocyte tissue volume in bone marrow is increased with aging and in patients with osteoporosis. Biogerontology.

[20] Lecka-Czernik B, Rosen CJ, Kawai M (2010). Skeletal aging and the adipocyte program: New insights from an “old” molecule. Cell Cycle.

[21] Meunier P, Aaron J, Edouard C, Vignon G (1971). Osteoporosis and the replacement of cell populations of the marrow by adipose tissue. A quantitative study of 84 iliac bone biopsies. Clin Orthop Relat Res.

[22] Ries L, Melbert D, Krapcho M, Stinchcomb D, Howlader N, Horner M, A M, Miller B, Feuer E, Altekruse S, Lewis D, Clegg L, Eisner M, Reichman M, BK E (2007). SEER Cancer Statistics Review, 1975-2005.

[23] Strotmeyer ES, Cauley JA (2007). Diabetes mellitus, bone mineral density, and fracture risk. Curr Opin Endocrinol Diabetes Obes.

[24] Bassett WW, Cooperberg MR, Sadetsky N, Silva S, DuChane J, Pasta DJ, Chan JM, Anast JW, Carroll PR, Kane CJ (2005). Impact of obesity on prostate cancer recurrence after radical prostatectomy: data from CaPSURE. Urology.

[25] Freedland SJ, Banez LL, Sun LL, Fitzsimons NJ, Moul JW (2009). Obese men have higher-grade and larger tumors: an analysis of the duke prostate center database. Prostate cancer and prostatic diseases.

[26] Gong Z, Neuhouser ML, Goodman PJ, Albanes D, Chi C, Hsing AW, Lippman SM, Platz EA, Pollak MN, Thompson IM, Kristal AR (2006). Obesity, diabetes, and risk of prostate cancer: results from the prostate cancer prevention trial. Cancer Epidemiol Biomarkers Prev.

[27] Scosyrev E, Messing EM, Mohile S, Golijanin D, Wu G (2012). Prostate cancer in the elderly: frequency of advanced disease at presentation and disease-specific mortality. Cancer.

[28] Keto CJ, Aronson WJ, Terris MK, Presti JC, Kane CJ, Amling CL, Freedland SJ (2012). Obesity is associated with castration-resistant disease and metastasis in men treated with androgen deprivation therapy after radical prostatectomy: results from the SEARCH database. BJU Int.

[29] Herroon MK, Rajagurubandara E, Hardaway AL, Powell K, Turchick A, Feldmann D, Podgorski I (2013). Bone marrow adipocytes promote tumor growth in bone *via* FABP4-dependent mechanisms. Oncotarget.

[30] Templeton ZS, Lie WR, Wang W, Rosenberg-Hasson Y, Alluri RV, Tamaresis JS, Bachmann MH, Lee K, Maloney WJ, Contag CH, King BL (2015). Breast Cancer Cell Colonization of the Human Bone Marrow Adipose Tissue Niche. Neoplasia.

[31] Martinez-Outschoorn UE, Sotgia F, Lisanti MP (2012). Power surge: supporting cells “fuel” cancer cell mitochondria. Cell metabolism.

[32] Schwartz B, Yehuda-Shnaidman E (2014). Putative role of adipose tissue in growth and metabolism of colon cancer cells. Front Oncol.

[33] Watson DG, Tonelli F, Alossaimi M, Williamson L, Chan E, Gorshkova I, Berdyshev E, Bittman R, Pyne NJ, Pyne S (2013). The roles of sphingosine kinases 1 and 2 in regulating the Warburg effect in prostate cancer cells. Cellular signalling.

[34] Tonelli F, Alossaimi M, Natarajan V, Gorshkova I, Berdyshev E, Bittman R, Watson DG, Pyne S, Pyne NJ (2013). The roles of sphingosine kinase 1 and 2 in regulating the metabolome and survival of prostate cancer cells. Biomolecules.

[35] Manzi L, Costantini L, Molinari R, Merendino N (2015). Effect of Dietary omega-3 Polyunsaturated Fatty Acid DHA on Glycolytic Enzymes and Warburg Phenotypes in Cancer. BioMed research international.

[36] Baenke F, Peck B, Miess H, Schulze A (2013). Hooked on fat: the role of lipid synthesis in cancer metabolism and tumour development. Dis Model Mech.

[37] Duncan RE, Ahmadian M, Jaworski K, Sarkadi-Nagy E, Sul HS (2007). Regulation of lipolysis in adipocytes. Annu Rev Nutr.

[38] Nieman KM, Kenny HA, Penicka CV, Ladanyi A, Buell-Gutbrod R, Zillhardt MR, Romero IL, Carey MS, Mills GB, Hotamisligil GS, Yamada SD, Peter ME, Gwin K, Lengyel E (2011). Adipocytes promote ovarian cancer metastasis and provide energy for rapid tumor growth. Nature medicine.

[39] Vaughan M (1962). The production and release of glycerol by adipose tissue incubated *in vitro*. J Biol Chem.

[40] Maeda N, Funahashi T, Shimomura I (2008). Metabolic impact of adipose and hepatic glycerol channels aquaporin 7 and aquaporin 9. Nature clinical practice Endocrinology & metabolism.

[41] Langin D (2006). Control of fatty acid and glycerol release in adipose tissue lipolysis. Comptes rendus biologies.

[42] Yamauchi T, Kamon J, Minokoshi Y, Ito Y, Waki H, Uchida S, Yamashita S, Noda M, Kita S, Ueki K, Eto K, Akanuma Y, Froguel P (2002). Adiponectin stimulates glucose utilization and fatty-acid oxidation by activating AMP-activated protein kinase. Nature medicine.

[43] Balaban S, Lee LS, Schreuder M, Hoy AJ (2015). Obesity and cancer progression: is there a role of fatty acid metabolism?. BioMed research international.

[44] Kroemer G, Pouyssegur J (2008). Tumor cell metabolism: cancer's Achilles’ heel. Cancer cell.

[45] Keith B, Johnson RS, Simon MC (2012). HIF1alpha and HIF2alpha: sibling rivalry in hypoxic tumour growth and progression. Nature reviews.

[46] Jochmanova I, Yang C, Zhuang Z, Pacak K (2013). Hypoxia-inducible factor signaling in pheochromocytoma: turning the rudder in the right direction. Journal of the National Cancer Institute.

[47] Philip B, Ito K, Moreno-Sanchez R, Ralph SJ (2013). HIF expression and the role of hypoxic microenvironments within primary tumours as protective sites driving cancer stem cell renewal and metastatic progression. Carcinogenesis.

[48] Raja R, Kale S, Thorat D, Soundararajan G, Lohite K, Mane A, Karnik S, Kundu GC (2014). Hypoxia-driven osteopontin contributes to breast tumor growth through modulation of HIF1alpha-mediated VEGF-dependent angiogenesis. Oncogene.

[49] Zecchini V, Madhu B, Russell R, Pertega-Gomes N, Warren A, Gaude E, Borlido J, Stark R, Ireland-Zecchini H, Rao R, Scott H, Boren J, Massie C (2014). Nuclear ARRB1 induces pseudohypoxia and cellular metabolism reprogramming in prostate cancer. EMBO J.

[50] Yehuda-Shnaidman E, Schwartz B (2012). Mechanisms linking obesity, inflammation and altered metabolism to colon carcinogenesis. Obes Rev.

[51] Cao JJ, Sun L, Gao H (2010). Diet-induced obesity alters bone remodeling leading to decreased femoral trabecular bone mass in mice. Ann N Y Acad Sci.

[52] Halade GV, El Jamali A, Williams PJ, Fajardo RJ, Fernandes G (2011). Obesity-mediated inflammatory microenvironment stimulates osteoclastogenesis and bone loss in mice. Exp Gerontol.

[53] Hardaway AL, Herroon MK, Rajagurubandara E, Podgorski I (2015). Marrow adipocyte-derived CXCL1 and CXCL2 contribute to osteolysis in metastatic prostate cancer. Clinical & experimental metastasis.

[54] Zhang J, Nuebel E, Wisidagama DR, Setoguchi K, Hong JS, Van Horn CM, Imam SS, Vergnes L, Malone CS, Koehler CM, Teitell MA (2012). Measuring energy metabolism in cultured cells, including human pluripotent stem cells and differentiated cells. Nature protocols.

[55] TeSlaa T, Teitell MA (2014). Techniques to monitor glycolysis. Methods Enzymol.

[56] Wu M, Neilson A, Swift AL, Moran R, Tamagnine J, Parslow D, Armistead S, Lemire K, Orrell J, Teich J, Chomicz S, Ferrick DA (2007). Multiparameter metabolic analysis reveals a close link between attenuated mitochondrial bioenergetic function and enhanced glycolysis dependency in human tumor cells. American journal of physiology Cell physiology.

[57] Haemmerle G, Lass A, Zimmermann R, Gorkiewicz G, Meyer C, Rozman J, Heldmaier G, Maier R, Theussl C, Eder S, Kratky D, Wagner EF, Klingenspor M, Hoefler G, Zechner R (2006). Defective lipolysis and altered energy metabolism in mice lacking adipose triglyceride lipase. Science.

[58] Haemmerle G, Moustafa T, Woelkart G, Buttner S, Schmidt A, van de Weijer T, Hesselink M, Jaeger D, Kienesberger PC, Zierler K, Schreiber R, Eichmann T, Kolb D (2011). ATGL-mediated fat catabolism regulates cardiac mitochondrial function *via* PPAR-alpha and PGC-1. Nature medicine.

[59] Mayer N, Schweiger M, Romauch M, Grabner GF, Eichmann TO, Fuchs E, Ivkovic J, Heier C, Mrak I, Lass A, Hofler G, Fledelius C, Zechner R, Zimmermann R, Breinbauer R (2013). Development of small-molecule inhibitors targeting adipose triglyceride lipase. Nature chemical biology.

[60] Zagani R, El-Assaad W, Gamache I, Teodoro JG (2015). Inhibition of adipose triglyceride lipase (ATGL) by the putative tumor suppressor G0S2 or a small molecule inhibitor attenuates the growth of cancer cells. Oncotarget.

[61] Furuhashi M, Hotamisligil GS (2008). Fatty acid-binding proteins: role in metabolic diseases and potential as drug targets. Nat Rev Drug Discov.

[62] Nomura DK, Long JZ, Niessen S, Hoover HS, Ng SW, Cravatt BF (2010). Monoacylglycerol lipase regulates a fatty acid network that promotes cancer pathogenesis. Cell.

[63] Yecies JL, Manning BD (2010). Chewing the fat on tumor cell metabolism. Cell.

[64] Ranasinghe WK, Baldwin GS, Shulkes A, Bolton D, Patel O (2014). Normoxic regulation of HIF-1alpha in prostate cancer. Nature reviews Urology.

[65] Semenza GL (2003). Targeting HIF-1 for cancer therapy. Nature reviews.

[66] Tokuda Y, Satoh Y, Fujiyama C, Toda S, Sugihara H, Masaki Z (2003). Prostate cancer cell growth is modulated by adipocyte-cancer cell interaction. BJU Int.

[67] Dirat B, Bochet L, Dabek M, Daviaud D, Dauvillier S, Majed B, Wang YY, Meulle A, Salles B, Le Gonidec S, Garrido I, Escourrou G, Valet P, Muller C (2011). Cancer-associated adipocytes exhibit an activated phenotype and contribute to breast cancer invasion. Cancer Res.

[68] Hefetz-Sela S, Scherer PE (2013). Adipocytes: impact on tumor growth and potential sites for therapeutic intervention. Pharmacology & therapeutics.

[69] Clark R, Krishnan V, Schoof M, Rodriguez I, Theriault B, Chekmareva M, Rinker-Schaeffer C (2013). Milky spots promote ovarian cancer metastatic colonization of peritoneal adipose in experimental models. Am J Pathol.

[70] Nieman K, Kenny H, Penicka C, Ladanyi A, Buell-Gutbrod R, Zillhardt M, Romero I, Carey M, Mills G, Hotamisligil G, Yamada S, Peter M, Gwin K, Lengyel E (2011). Adipocytes promote ovarian cancer metastasis and provide energy for rapid tumor growth. Nature medicine.

[71] Tan J, Buache E, Chenard MP, Dali-Youcef N, Rio MC (2011). Adipocyte is a non-trivial, dynamic partner of breast cancer cells. Int J Dev Biol.

[72] Wang C, Gao C, Meng K, Qiao H, Wang Y (2015). Human adipocytes stimulate invasion of breast cancer MCF-7 cells by secreting IGFBP-2. PLoS ONE.

[73] Ribeiro R, Monteiro C, Cunha V, Oliveira MJ, Freitas M, Fraga A, Principe P, Lobato C, Lobo F, Morais A, Silva V, Sanches-Magalhaes J, Oliveira J (2012). Human periprostatic adipose tissue promotes prostate cancer aggressiveness *in vitro*. J Exp Clin Cancer Res.

[74] Fang JS, Gillies RD, Gatenby RA (2008). Adaptation to hypoxia and acidosis in arcinogenesis and tumor progression. Semin Cancer Biol.

[75] Chiche J, Brahimi-Horn MC, Pouyssegur J (2010). Tumour hypoxia induces a metabolic shift causing acidosis: a common feature in cancer. J Cell Mol Med.

[76] Elstrom RL, Bauer DE, Buzzai M, Karnauskas R, Harris MH, Plas DR, Zhuang H, Cinalli RM, Alavi A, Rudin CM, Thompson CB (2004). Akt stimulates aerobic glycolysis in cancer cells. Cancer Res.

[77] Lord-Fontaine S, Averill-Bates DA (2002). Heat shock inactivates cellular antioxidant defenses against hydrogen peroxide: protection by glucose. Free Radic Biol Med.

[78] Sullivan R, Pare GC, Frederiksen LJ, Semenza GL, Graham CH (2008). Hypoxia-induced resistance to anticancer drugs is associated with decreased senescence and requires hypoxia-inducible factor-1 activity. Mol Cancer Ther.

[79] Doktorova H, Hrabeta J, Khalil MA, Eckschlager T (2015). Hypoxia-induced chemoresistance in cancer cells: The role of not only HIF-1. Biomedical papers of the Medical Faculty of the University Palacky, Olomouc, Czechoslovakia.

[80] Yoshida GJ (2015). Metabolic reprogramming: the emerging concept and associated therapeutic strategies. J Exp Clin Cancer Res.

[81] Liu L, Ning X, Sun L, Zhang H, Shi Y, Guo C, Han S, Liu J, Sun S, Han Z, Wu K, Fan D (2008). Hypoxia-inducible factor-1 alpha contributes to hypoxia-induced chemoresistance in gastric cancer. Cancer science.

[82] Lu H, Forbes RA, Verma A (2002). Hypoxia-inducible factor 1 activation by aerobic glycolysis implicates the Warburg effect in carcinogenesis. J Biol Chem.

[83] Marin-Hernandez A, Gallardo-Perez JC, Ralph SJ, Rodriguez-Enriquez S, Moreno-Sanchez R (2009). HIF-1alpha modulates energy metabolism in cancer cells by inducing over-expression of specific glycolytic isoforms. Mini reviews in medicinal chemistry.

[84] Huang SW, Kao JK, Wu CY, Wang ST, Lee HC, Liang SM, Chen YJ, Shieh JJ (2014). Targeting aerobic glycolysis and HIF-1alpha expression enhance imiquimod-induced apoptosis in cancer cells. Oncotarget.

[85] Kim JW, Tchernyshyov I, Semenza GL, Dang CV (2006). HIF-1-mediated expression of pyruvate dehydrogenase kinase: a metabolic switch required for cellular adaptation to hypoxia. Cell metabolism.

[86] Papandreou I, Cairns RA, Fontana L, Lim AL, Denko NC (2006). HIF-1 mediates adaptation to hypoxia by actively downregulating mitochondrial oxygen consumption. Cell metabolism.

[87] Sonveaux P, Vegran F, Schroeder T, Wergin MC, Verrax J, Rabbani ZN, De Saedeleer CJ, Kennedy KM, Diepart C, Jordan BF, Kelley MJ, Gallez B, Wahl ML, Feron O, Dewhirst MW (2008). Targeting lactate-fueled respiration selectively kills hypoxic tumor cells in mice. J Clin Invest.

[88] Morgan PE, Pastorekova S, Stuart-Tilley AK, Alper SL, Casey JR (2007). Interactions of transmembrane carbonic anhydrase, CAIX, with bicarbonate transporters. American journal of physiology Cell physiology.

[89] Asosingh K, De Raeve H, de Ridder M, Storme GA, Willems A, Van Riet I, Van Camp B, Vanderkerken K (2005). Role of the hypoxic bone marrow microenvironment in 5T2MM murine myeloma tumor progression. Haematologica.

[90] Nombela-Arrieta C, Pivarnik G, Winkel B, Canty KJ, Harley B, Mahoney JE, Park SY, Lu J, Protopopov A, Silberstein LE (2013). Quantitative imaging of haematopoietic stem and progenitor cell localization and hypoxic status in the bone marrow microenvironment. Nature cell biology.

[91] Spencer JA, Ferraro F, Roussakis E, Klein A, Wu J, Runnels JM, Zaher W, Mortensen LJ, Alt C, Turcotte R, Yusuf R, Cote D, Vinogradov SA, Scadden DT, Lin CP (2014). Direct measurement of local oxygen concentration in the bone marrow of live animals. Nature.

[92] Bratslavsky G, Sudarshan S, Neckers L, Linehan WM (2007). Pseudohypoxic pathways in renal cell carcinoma. Clin Cancer Res.

[93] Guzzo G, Sciacovelli M, Bernardi P, Rasola A (2014). Inhibition of succinate dehydrogenase by the mitochondrial chaperone TRAP1 has anti-oxidant and anti-apoptotic effects on tumor cells. Oncotarget.

[94] Aragones J, Fraisl P, Baes M, Carmeliet P (2009). Oxygen sensors at the crossroad of metabolism. Cell metabolism.

[95] Frezza C, Pollard PJ, Gottlieb E (2011). Inborn and acquired metabolic defects in cancer. Journal of molecular medicine.

[96] Bensaad K, Favaro E, Lewis CA, Peck B, Lord S, Collins JM, Pinnick KE, Wigfield S, Buffa FM, Li JL, Zhang Q, Wakelam MJ, Karpe F, Schulze A, Harris AL (2014). Fatty acid uptake and lipid storage induced by HIF-1alpha contribute to cell growth and survival after hypoxia-reoxygenation. Cell reports.

[97] Ackerman D, Simon MC (2014). Hypoxia, lipids, and cancer: surviving the harsh tumor microenvironment. Trends Cell Biol.

[98] Kamphorst JJ, Cross JR, Fan J, de Stanchina E, Mathew R, White EP, Thompson CB, Rabinowitz JD (2013). Hypoxic and Ras-transformed cells support growth by scavenging unsaturated fatty acids from lysophospholipids. Proc Natl Acad Sci U S A.

[99] Nomura DK, Lombardi DP, Chang JW, Niessen S, Ward AM, Long JZ, Hoover HH, Cravatt BF (2011). Monoacylglycerol lipase exerts dual control over endocannabinoid and fatty acid pathways to support prostate cancer. Chem Biol.

[100] Pedersen PL, Mathupala S, Rempel A, Geschwind JF, Ko YH (2002). Mitochondrial bound type II hexokinase: a key player in the growth and survival of many cancers and an ideal prospect for therapeutic intervention. Biochim Biophys Acta.

[101] Nakashima RA, Mangan PS, Colombini M, Pedersen PL (1986). Hexokinase receptor complex in hepatoma mitochondria: evidence from N,N’-dicyclohexylcarbodiimide-labeling studies for the involvement of the pore-forming protein VDAC. Biochemistry.

[102] Mathupala SP, Ko YH, Pedersen PL (2006). Hexokinase II: cancer's double-edged sword acting as both facilitator and gatekeeper of malignancy when bound to mitochondria. Oncogene.

[103] Mathupala SP, Ko YH, Pedersen PL (2009). Hexokinase-2 bound to mitochondria: cancer's stygian link to the “Warburg Effect” and a pivotal target for effective therapy. Semin Cancer Biol.

[104] Marrache S, Dhar S (2015). The energy blocker inside the power house: Mitochondria targeted delivery of 3-bromopyruvate. Chemical science.

[105] Chiara F, Castellaro D, Marin O, Petronilli V, Brusilow WS, Juhaszova M, Sollott SJ, Forte M, Bernardi P, Rasola A (2008). Hexokinase II detachment from mitochondria triggers apoptosis through the permeability transition pore independent of voltage-dependent anion channels. PLoS ONE.

[106] Pastorino JG, Hoek JB (2003). Hexokinase II: the integration of energy metabolism and control of apoptosis. Curr Med Chem.

[107] Arora KK, Pedersen PL (1988). Functional significance of mitochondrial bound hexokinase in tumor cell metabolism. Evidence for preferential phosphorylation of glucose by intramitochondrially generated ATP. J Biol Chem.

[108] Brisset JC, Hoff BA, Chenevert TL, Jacobson JA, Boes JL, Galban S, Rehemtulla A, Johnson TD, Pienta KJ, Galban CJ, Meyer CR, Schakel T, Nicolay K, Alva AS, Hussain M, Ross BD (2015). Integrated multimodal imaging of dynamic bone-tumor alterations associated with metastatic prostate cancer. PLoS ONE.

[109] Cunningham D, You Z (2015). *In vitro* and *in vivo* model systems used in prostate cancer esearch. Journal of biological methods.

[110] Park SI, Kim SJ, McCauley LK, Gallick GE (2010). Pre-clinical mouse models of human prostate cancer and their utility in drug discovery. Current protocols in pharmacology.

[111] Chen GL, Luo Y, Eriksson D, Meng X, Qian C, Bauerle T, Chen XX, Schett G, Bozec A (2016). High fat diet increases melanoma cell growth in the bone marrow by inducing osteopontin and interleukin 6. Oncotarget.

[112] Shankar E, Vykhovanets EV, Vykhovanets OV, Maclennan GT, Singh R, Bhaskaran N, Shukla S, Gupta S (2012). High-fat diet activates pro-inflammatory response in the prostate through association of Stat-3 and NF-kappaB. Prostate.

[113] Suburu J, Chen YQ (2012). Lipids and prostate cancer. Prostaglandins Other Lipid Mediat.

[114] Boroughs LK, DeBerardinis RJ (2015). Metabolic pathways promoting cancer cell survival and growth. Nature cell biology.

[115] Pastorino JG, Shulga N, Hoek JB (2002). Mitochondrial binding of hexokinase II inhibits Bax-induced cytochrome c release and apoptosis. J Biol Chem.

[116] Wang Z, Liu P, Chen Q, Deng S, Liu X, Situ H, Zhong S, Hann S, Lin Y (2015). Targeting AMPK signaling pathway to overcome drug resistance for cancer therapy. Curr Drug Targets.

[117] Derdak Z, Mark NM, Beldi G, Robson SC, Wands JR, Baffy G (2008). The mitochondrial uncoupling protein-2 promotes chemoresistance in cancer cells. Cancer Res.

[118] Guaragnella N, Giannattasio S, Moro L (2014). Mitochondrial dysfunction in cancer chemoresistance. Biochem Pharmacol.

[119] Kaighn ME, Narayan KS, Ohnuki Y, Lechner JF, Jones LW (1979). Establishment and characterization of a human prostatic carcinoma cell line (PC-3). Investigative urology.

[120] Stone KR, Mickey DD, Wunderli H, Mickey GH, Paulson DF (1978). Isolation of a human prostate carcinoma cell line (DU 145). Int J Cancer.

[121] Zhau HE, Odero-Marah V, Lue HW, Nomura T, Wang R, Chu G, Liu ZR, Zhou BP, Huang WC, Chung LW (2008). Epithelial to mesenchymal transition (EMT) in human prostate cancer: lessons learned from ARCaP model. Clinical & experimental metastasis.

[122] Podgorski I, Linebaugh BE, Koblinski JE, Rudy DL, Herroon MK, Olive MB, Sloane BF (2009). Bone marrow-derived cathepsin K cleaves SPARC in bone metastasis. Am J Pathol.

[123] Herroon MK, Rajagurubandara E, Rudy DL, Chalasani A, Hardaway AL, Podgorski I (2013). Macrophage cathepsin K promotes prostate tumor progression in bone. Oncogene.

[124] Lee I, Pecinova A, Pecina P, Neel BG, Araki T, Kucherlapati R, Roberts AE, Huttemann M (2010). A suggested role for mitochondria in Noonan syndrome. Biochim Biophys Acta.

[125] Salvioli S, Ardizzoni A, Franceschi C, Cossarizza A (1997). JC-1, but not DiOC6(3) or rhodamine 123, is a reliable fluorescent probe to assess delta psi changes in intact cells: implications for studies on mitochondrial functionality during apoptosis. FEBS Lett.

