# Supplementary material for: Bone marrow adipocytes promote the Warburg phenotype in metastatic prostate tumors via HIF-1α activation
Source: Oncotarget. 2016 Aug 30;7(40):64854–77. doi: 10.18632/oncotarget.11712 (PMC5323121; doi:10.18632/oncotarget.11712)
Supplement: Supplementary file 1 [file oncotarget-07-64854-s001.pdf]

Bone marrow adipocytes promote the warburg phenotype in metastatic prostate tumors *via* HIF-1α activation

Supplementary Material

Supplementary Table 1: Oncomine gene analysis of 13 prostate datasets comparing upregulated genes involved in glycolysis and hypoxic response in prostate cancer patients with primary and metastatic disease. *Enolase* (*ENO2*), *hexokinase 2* (*HK2*), and *glucose transporter 1* (*GLUT1*) were upregulated in 3/13, 4/9, and 5/13 available datasets, respectively. Warburg-associated enzymes *lactate dehydrogenase* (*LDHa*) and *pyruvate dehydrogenase kinase 1* (*PDK1)* were upregulated in metastatic sites of patients compared to primary prostate cancer in 8/13 and 8/12 of the datasets, respectively. Hypoxic responsive genes *carbonic anhydrase 9* (*CA9*) and *vascular endothelial growth factor alpha* (*VEGFA*) were upregulated in 6/12 and 5/13 metastatic tumors when compared to primary tumors. (n = number of samples; P = Primary site; M = Metastatic site).

Gene	Prostate Cancer	Fold Change	P Value	n
ENO2 (3/13)	Grasso (126)	2.22	4.33E-4	P: 59; M: 35
	LaTulippe (127)	1.45	0.034	P: 23; M: 9
	Varambally (128)	6.122	0.009	P: 7; M: 6
LDHa (8/13)	Grasso (126)	1.60	0.001	P: 59; M: 35
	LaTulippe (127)	1.78	0.008	P: 23; M: 9
	Holzbeierlein (129)	1.73	0.009	P: 40; M: 9
	Chandran (130)	1.68	0.003	P: 10; M: 21
	Ramaswamy 2 (131)	3.06	0.003	P: 10; M: 3
	Ramaswamy (132)	2.60	0.009	P: 10; M: 4
	Yu (133)	3.052	2.47E-7	P: 64; M: 24
	Varambally (128)	2.176	7.25E-4	P: 7; M: 6
PDK1 (8/12)	Grasso (126)	2.96	1.27E-7	P: 59; M: 35
	LaTulippe (127)	2.78	0.005	P: 23; M: 9
	Chandran	1.44	0.002	P: 10; M: 21
	(130)(129)(129)(129)(129)(129)	1.63	0.015	P: 62; M: 9
	Lapointe (134)	1.114	0.005	P: 27; M: 5
	Vanaja (135)	1.370	9.99E-8	P: 64; M: 24
	Yu (133)	2.152	0.021	P: 23; M: 12
	Tamura (136)	2.167	0.030	P: 7; M: 6
	Varambally (128)			
HK2 (4/9)	Grasso (126)	4.755	3.53E-9	P: 59; M: 35
	Varambally (128)	4.427	4.59E-4	P: 7; M: 6
	Ramaswamy 2 (131)	9.507	0.021	P: 10; M: 3
	Chandran (130)	3.291	3.40E-6	P: 10; M: 21
GLUT1 (5/13)	Varambally(128)	1.633	5.74E-5	P: 7; M: 6
	Ramaswamy(132)	2.049	0.017	P: 10; M: 4
	Ramaswamy 2(131)	2.132	0.019	P: 10; M: 3
	Yu (133)	1.088	4.63E-4	P: 64; M: 24
	Grasso (126)	2.048	1.51E-5	P: 59; M: 35
CA9 (6/12)	Ramaswamy (132)	10.458	0.010	P: 10; M: 4
	Ramaswamy 2 (131)	11.031	0.010	P: 10; M: 3
	Varambally (128)	3.131	0.003	P: 7; M: 6
	Yu (133)	1.109	0.003	P: 64; M: 24
	Grasso (126)	3.363	4.79E-6	P: 59; M: 35
	Chandran (130)	1.487	1.22E-4	P: 10; M: 21
VEGFA (5/13)	Grasso (126)	7.552	1.55E-14	P: 59; M: 35

	Varambally (128)<br>Tamura (136)<br>Yu (133)<br>Chandran (130)	3.750<br>2.443<br>1.594<br>2.488	1.10E-5<br>0.014<br>5.14E-5<br>9.27E-4	P: 7; M: 6<br>P: 23; M: 12<br>P: 64; M: 24<br>P: 10; M: 21

Supplementary Table 2: Oncomine gene analysis of Grasso Prostate database comparing upregulated genes involved in glycolysis in prostate cancer patients with primary and metastatic disease. Significantly upregulated genes are shown with a p value of $p < 0.05$.

GENE	FOLD CHANGE	P-VALUE
**Triosephosphate Isomerase 1 (TPI1)**	2.31	2.30E-13
**Pyruvate Kinase M2 (PKM2)**	2.67	1.47E-9
**Hexokinase 2 (HK2)**	4.75	3.53E-9
**Enolase 3 (ENO3)**	2.85	1.05E-8
**Glucose-6-Phosphate Isomerase (GPI)**	2.01	1.43E-8
**Aldolase C (ALDOC)**	2.69	2.41E-8
**Dihydrolipoamide 5-Acetyltransferase (DLAT)**	1.95	3.02E-8
**Phosphoglycerate Kinase 1 (PGK1)**	6.77	8.17E-8
**Aldolase A (ALDOA)**	1.85	1.16E-7
**Transmembrane Protein 54 (TMEM54)**	1.99	3.10E-7
**Pyruvate Dehydrogenase Alpha 1 (PDHA1)**	1.69	6.20E-7
**Aldolase B (ALDOB)**	12.85	1.99E-6
**Oxoglutarate Dehydrogenase (OGDH)**	1.55	1.84E-5
**Phosphoglycerate Mutase 1 (PGAM1)**	1.69	2.12E-5
**Hexokinase Domain Containing 1 (HKDC1)**	2.37	1.23E-4
**Glyceraldehyde-3-Phosphate Dehydrogenase (GAPDH)**	1.57	2.63E-4
**Enolase 2 (ENO2)**	2.22	4.33E-4
**Phosphoglycerate Mutase 2 (PGAM2)**	1.79	4.99E-4
**Lactate Dehydrogenase Alpha (LDHA)**	1.60	0.001
**Phosphofructokinase, Platelet (PFKP)**	1.63	0.001
**Hexokinase 3 (HK3)**	1.45	0.004
**IQ Motif Containing D (IQCD)**	1.56	0.004
**Pyruvate Kinase, Liver and RBC (PKLR)**	1.29	0.013
**Glucokinase (GCK)**	1.28	0.020
**Glyceraldehyde-3-PhosphateDehydrogenase, Spermatogenic (GAPDHS)**	1.68	0.021
**Lactate Dehydrogenase A-Like 6A (LDHAL6A)**	1.64	0.023

Supplementary Table 3: Oncomine gene analysis of Grasso Prostate database comparing upregulated genes involved in HIF-1α activation in prostate cancer patients with primary and metastatic disease. Significantly upregulated genes are shown with a p value of $p < 0.05$.

GENE	FOLD CHANGE	P-VALUE
**Stanniocalcin 2 (STC2)**	7.33	3.96E-15
**Vascular Endothelial Growth Factor Alpha (VEGFA)**	7.55	1.55E-14
**Solute Carrier Family 6 (SLC6A8)**	2.35	1.45E-12
**Short Stature Homeobox 2 (SHOX2)**	6.14	2.33E-12
**Kruppel-Like Factor 11 (KLF11)**	2.72	3.69E-12
**Sperm Associated Antigen 4 (SPAG4)**	4.66	8.30E-12
**SRY-Box-4 (SOX4)**	4.47	2.90E-11
**EGL Nine Homolog 1 (EGLN1)**	2.44	3.15E-11
**Notch Homolog 4 (NOTCH4)**	2.08	3.25E-11
**Adenosine A2 Alpha Receptor (ADORA2A)**	2.20	5.82E-11
**DNA-Damage-Inducible Transcript 4 (DDIT4)**	4.38	9.58E-11
**Family With Sequence Similarity 128, Member B (FAM128B)**	2.50	1.18E-10
**Tropomysoin 1 (TPM1)**	3.83	1.27E-10
**Triple Functional Domain (TRIO)**	3.01	1.52E-10
**Procollagen-Lysine, 2-Oxoglutarate 5-dioxygenase 2 (PLOD2)**	4.26	5.94E-10
**Zinc Finger Protein 292 (ZNF292)**	4.70	6.22E-10
**Platelet Derived Growth Factor B (PDGFB)**	2.36	1.65E-8
**Biglycan (BGN)**	1.88	2.32E-8
**Tumor Necrosis Factor Receptor Superfamily, Member 10B (TNFRSF10B)**	2.55	2.39E-8
**Nuclear Prelamain A Recognition Factor (NARF)**	2.07	2.37E-8
**Collagen, Type IV, Alpha 1 (COL4A1)**	2.87	2.90E-8
**Angiopoietin-Like 4 (ANGPTL4)**	4.33	4.30E-8
**EGL Nine Homolog 3 (EGLN3)**	4.10	5.56E-8
**Discs, Large Homolog-Associated Protein 4 (DLGAP4)**	2.19	6.89E-8
**Coagulation Factor VIII (F8)**	3.16	1.26E-7
**Cyclin Dependent Kinase 19 (CDK19)**	2.45	1.60E-7

Supplementary Table 4. Specificity of human Taqman probes utilized in *in vivo studies*. Transcript levels for human glycolysis- and hypoxia-associated genes were determined in murine bone tissues and murine adipocytes. Samples were run in triplicate and CT values >35 were considered as 'Undetermined'. Among all probes tested, only ENO2 showed very small cross-reactivity with mouse samples.

GENE	Probe ID	CT values (in murine samples)
HPRT1	Hs02800695	Undetermined
GLUT1	Hs00892681	Undetermined
ENO2	Hs00157360	33.1 ± 0.15
PDK1	Hs01561850	Undetermined
LDHA	Hs00855322	Undetermined
HK2	Hs00606086	Undetermined
CA9	Hs00154208	Undetermined
VEGF	Hs00900055	Undetermined

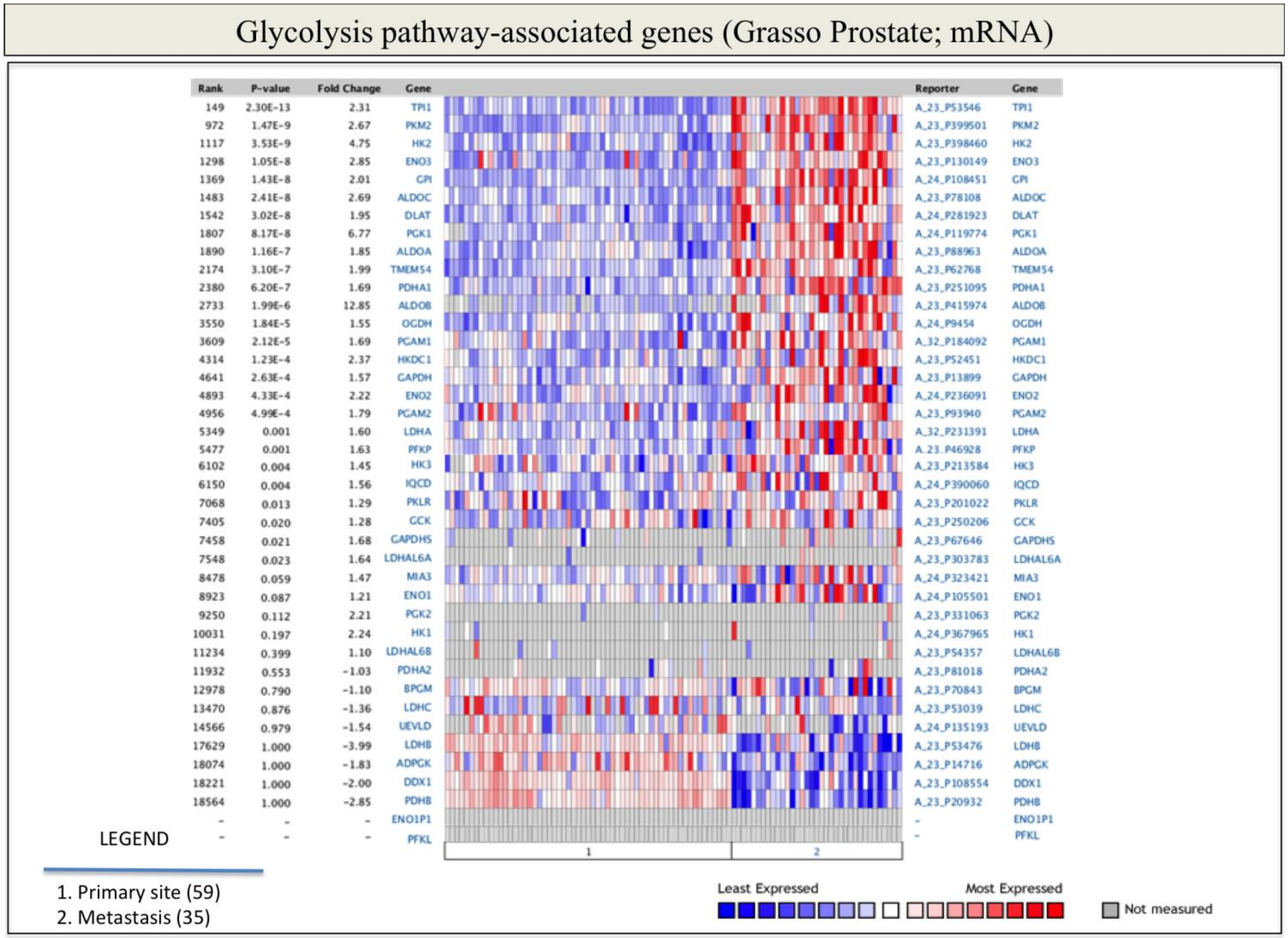

Supplementary Figure 1. Oncomine analysis of Grasso prostate dataset comparing expression of glycolysis-associated genes in primary (P) and metastatic (M) prostate cancers. Heat plot of significantly altered genes. Data were ordered by ‘overexpression’ and the threshold was adjusted to P-value <1E-4; fold change, 2 and gene rank, top 10%.

Cbioportal analysis of glycolysis-associated genes in prostate cancer

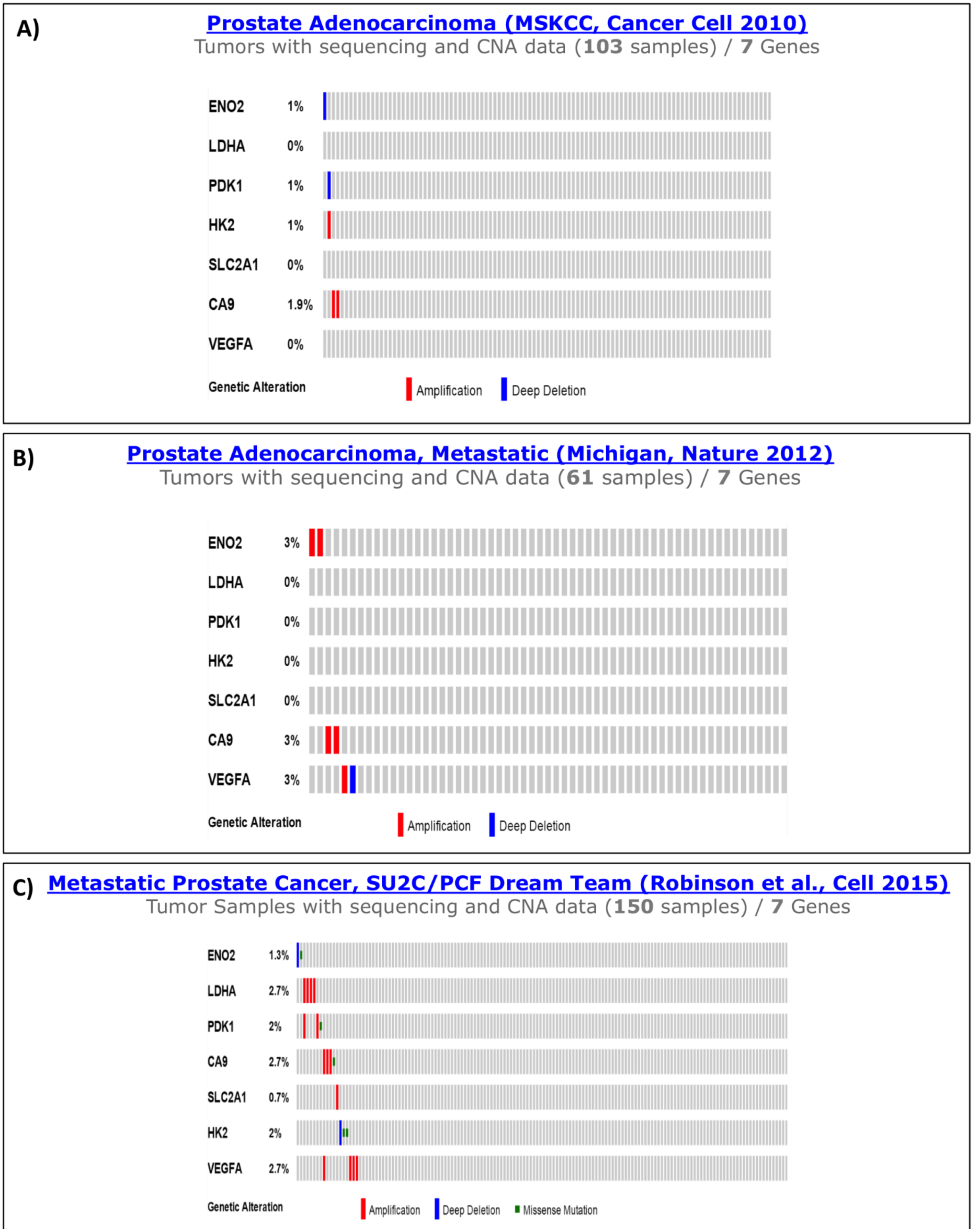

Supplementary Figure 2: CBioPortal analysis of genetic copy number alterations (CNA) and mutations in glycolysis associated genes *enolase* (*ENO2*), *lactate dehydrogenase alpha* (*LDHa*), *pyruvate dehydrogenase kinase 1* (*PDK1*), *hexokinase 2* (*HK2*), and *glucose transporter 1* (*SLC2A1*), and hypoxic response genes *carbonic anhydrase 9* (*CA9*) and *vascular endothelial growth factor A* (*VEGFA*) across three different datasets of metastatic prostate cancer patients (126, 137-140). Vertical bars represent individual patients. If there are genetic alterations within the gene of interest, a red bar represents amplification of the gene, blue bars depict deep deletions within the gene, and green bars signify mutations within the gene. Gray bars represent unaffected patients.

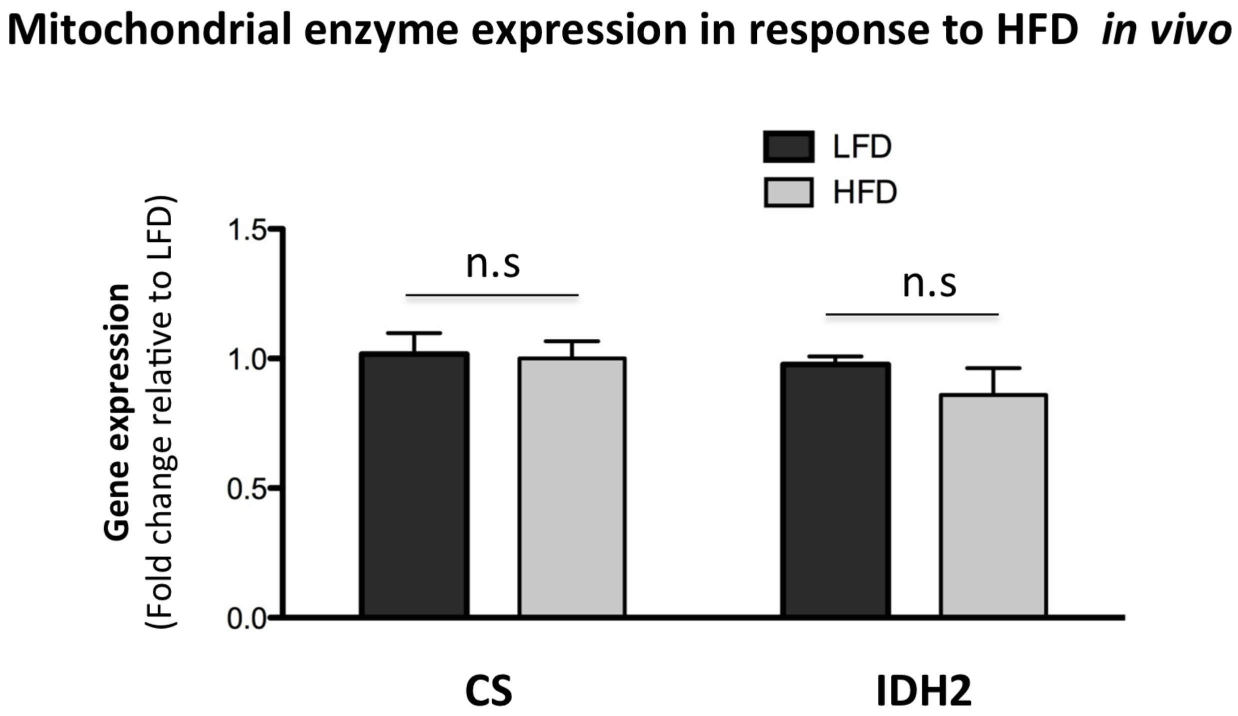

Supplementary Figure 3: Expression of mitochondrial enzymes in intratibial prostate tumors is not affected by HFD-induced marrow adiposity. Taqman RT-PCR analysis of *citrate synthase* (*CS*) and *isocitrate dehydrogenase 2* (*IDH2*) in PC3 bone tumors. Data are normalized to *EPCAM* and shown as increase relative to control. Results represent a mean of at least 3 independent experiments ± SD.

Expression of glycolytic genes in bone tumors after HFD to LFD switch

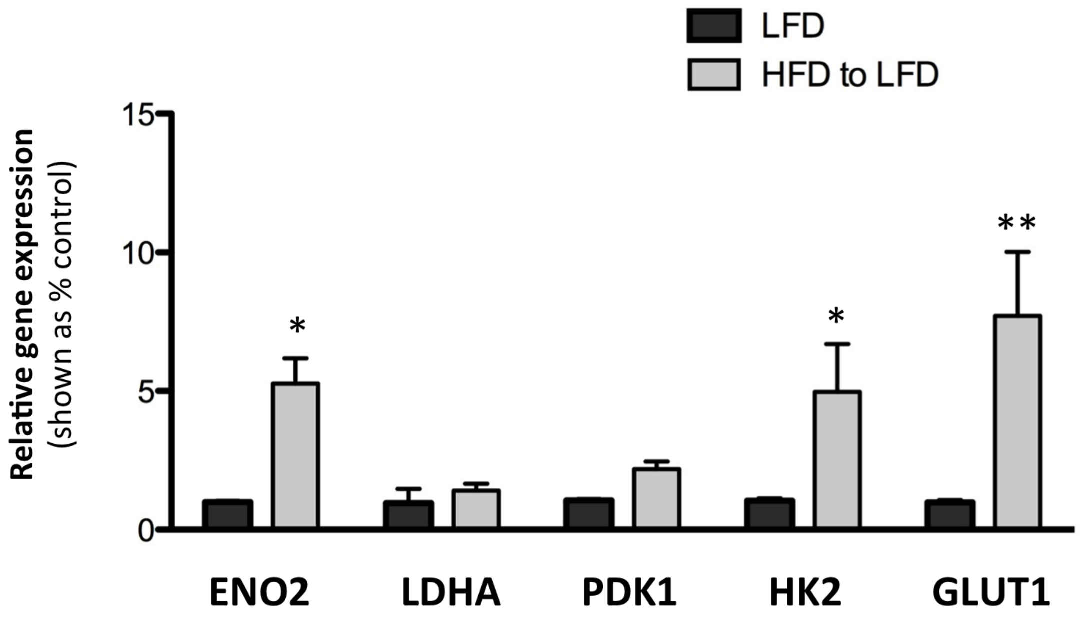

Supplementary Figure 4: Comparative RT-PCR analysis of Warburg Effect-associated genes in bone tumors from mice on LFD vs. mice with HFD-induced marrow adiposity that were switched to LFD upon tumor implantation into the tibia (HFD to LFD). Data, showing persisting glycolytic phenotype in 'HFD to LFD' mice were normalized to human *EPCAM* and represent a mean of a minimum of 3 mice/group ± SD. Values * $p < 0.05$; ** $p < 0.01$ are considered statistically significant.

Expression of glycolytic genes upon co-culture with bone marrow stromal cells

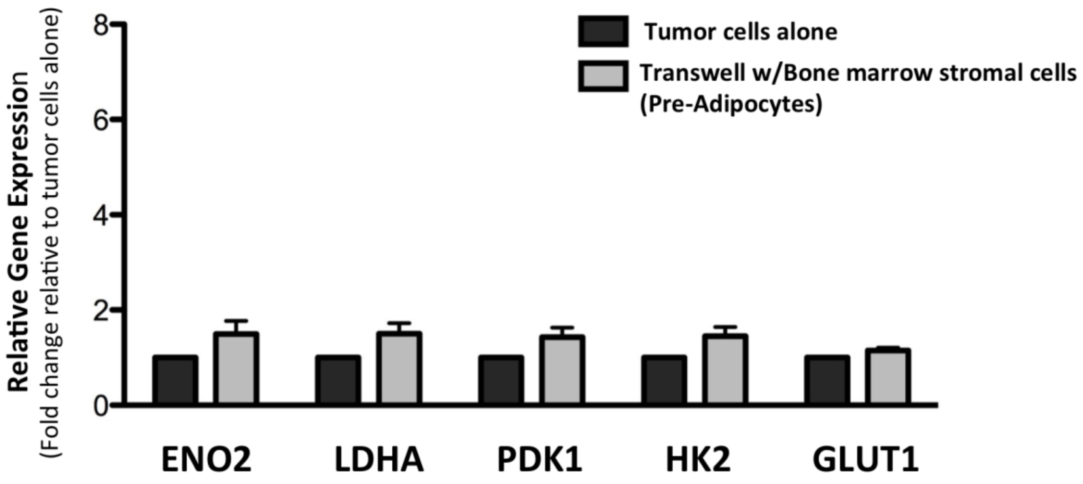

Supplementary Figure 5. Bone marrow stromal cells (pre-adipocytes) do not induce Warburg Effect-associated genes. Taqman RT-PCR analysis of *ENO2*, *LDHa*, *PDK1*, *HK2*, and *GLUT1* in PC3 cells grown in transwell co-culture with pre-adipocytes. Data are normalized to *HPRT1* and shown as increase relative to control. Results represent a mean of at least 3 independent experiments ± SD.

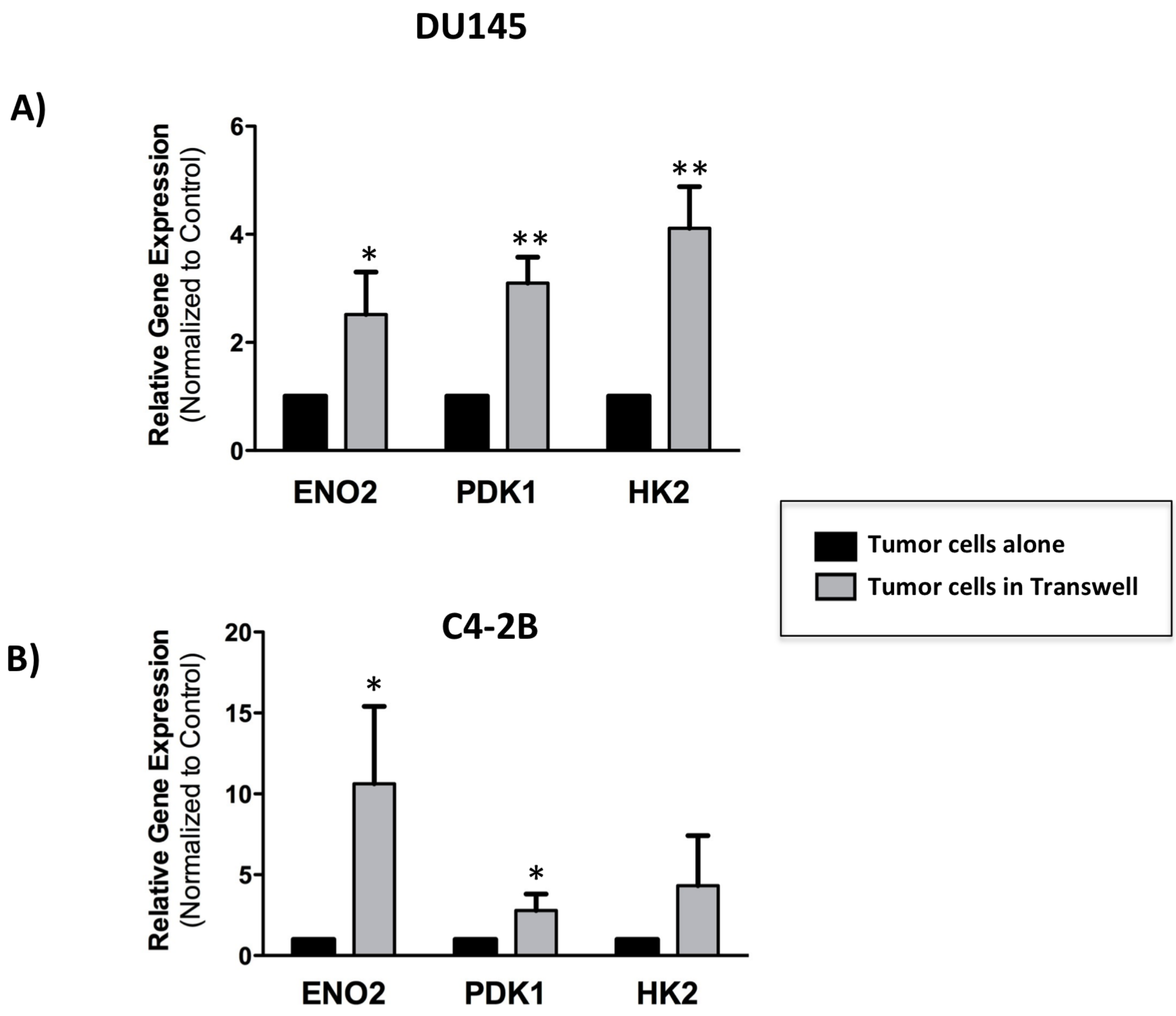

Supplementary Figure 6. Bone marrow adipocytes enhance Warburg Effect in DU145 and C4-2B cells. Taqman RT-PCR analysis of *ENO2*, *PDK1*, and *HK2* in DU145 (top) and C4-2B (bottom) cells cultured alone or in transwell co-culture with bone marrow adipocytes. Data are normalized to *HPRT1* and shown as increase relative to control. Results represent a mean of at least 3 independent experiments ± SD. Values * $p < 0.05$; ** $p < 0.001$ are considered statistically significant.

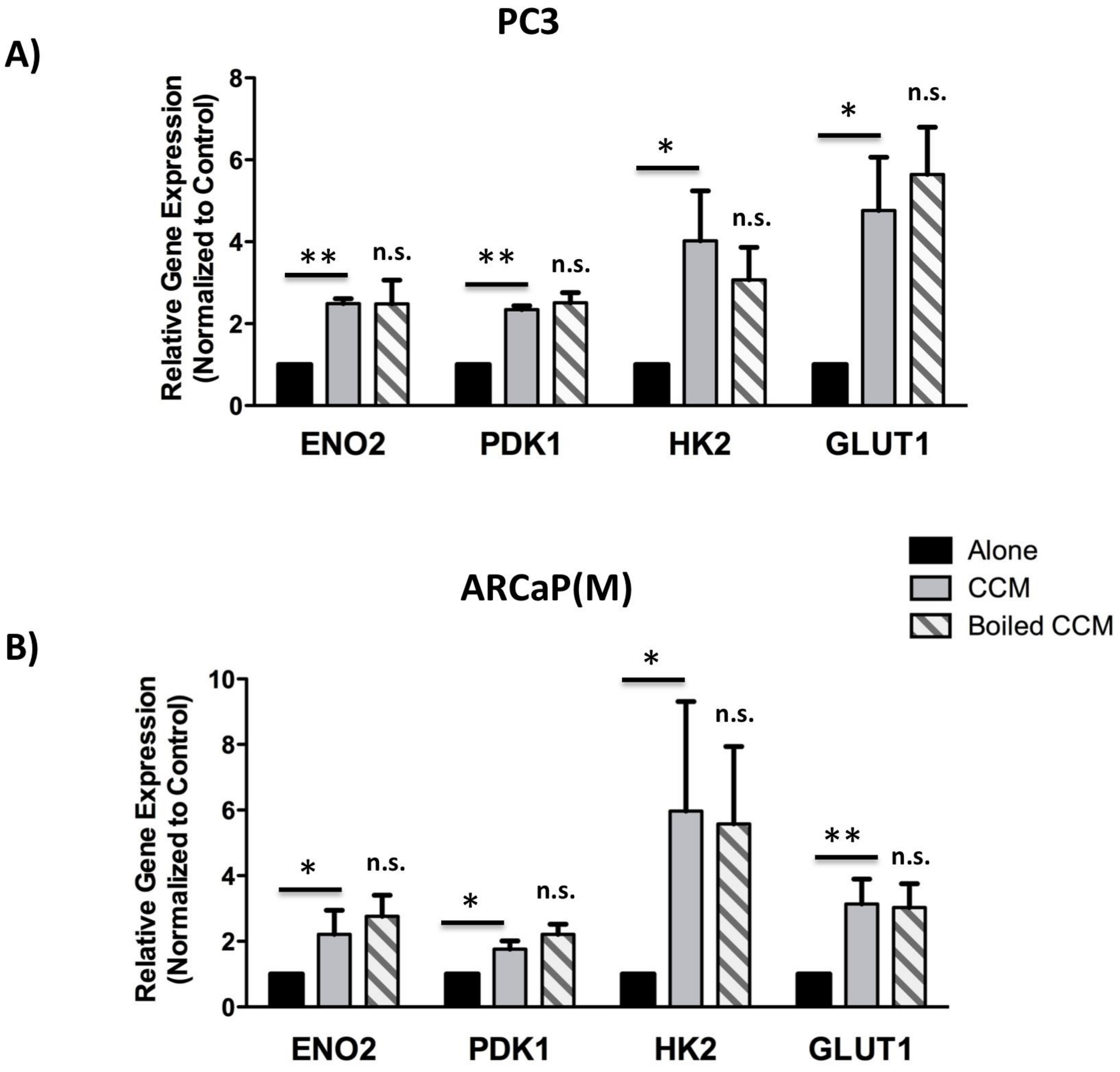

Supplementary Figure 7. Warburg phenotype in prostate cancer cells is driven by adipocyte-derived lipids. Taqman RT-PCR analysis of *ENO2*, *PDK1*, *HK2*, and *GLUT1* in PC3 (top) and ARCaP(M) (bottom) in the presence or absence of co-culture conditioned media (CCM) and boiled co-culture conditioned media (boiled CCM). Data are normalized to *HPRT1* and shown as increase relative to control. Results represent a mean of at least 3 independent experiments ± SD. Values * $p < 0.05$; ** $p < 0.01$ are considered statistically significant.

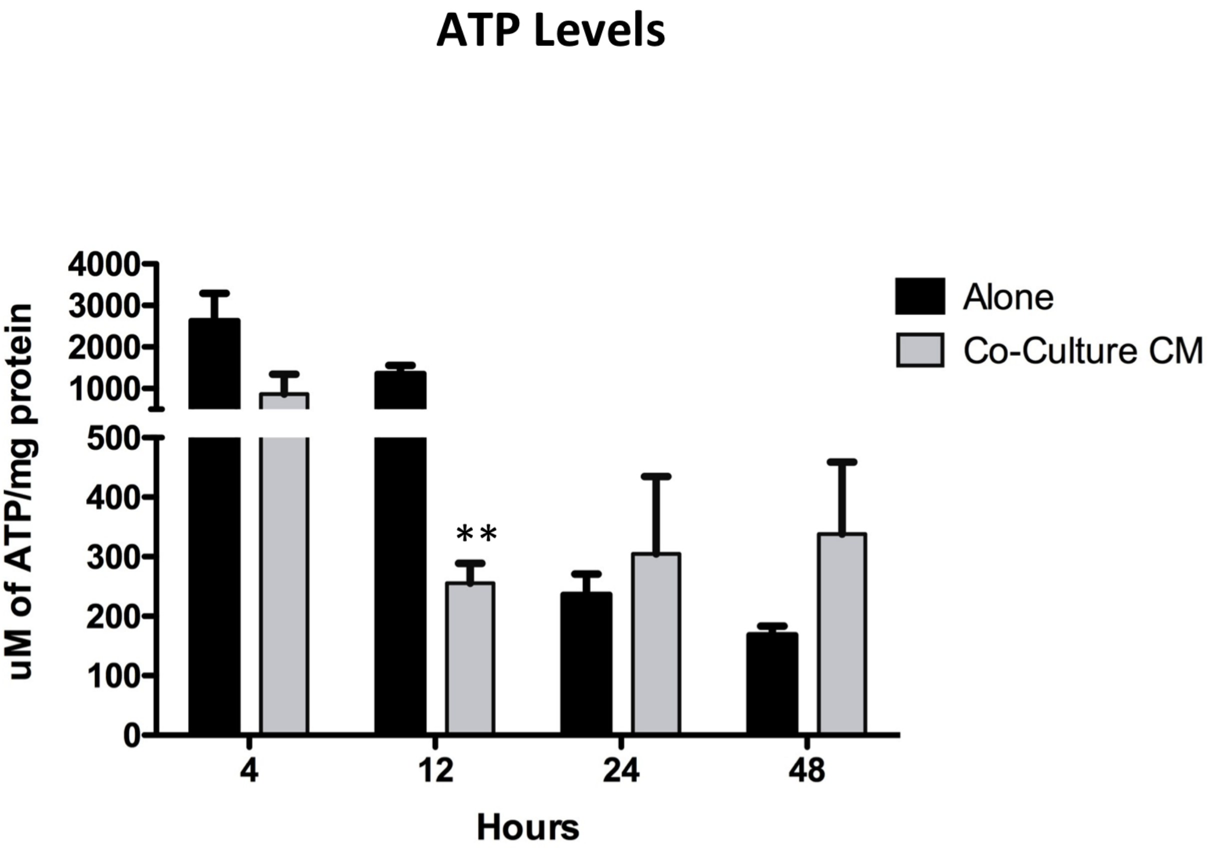

Supplementary Figure 8. Changes in ATP levels with time are indicative of glycolytic flux. ATP levels in PC3 cells cultured in the presence or absence of CCM for 4-48 hours. Significant decrease in ATP levels was observed after 12 hours in CCM with evidence of recovery after 24 and 48 hours. Values ** $p < 0.01$ are considered statistically significant.

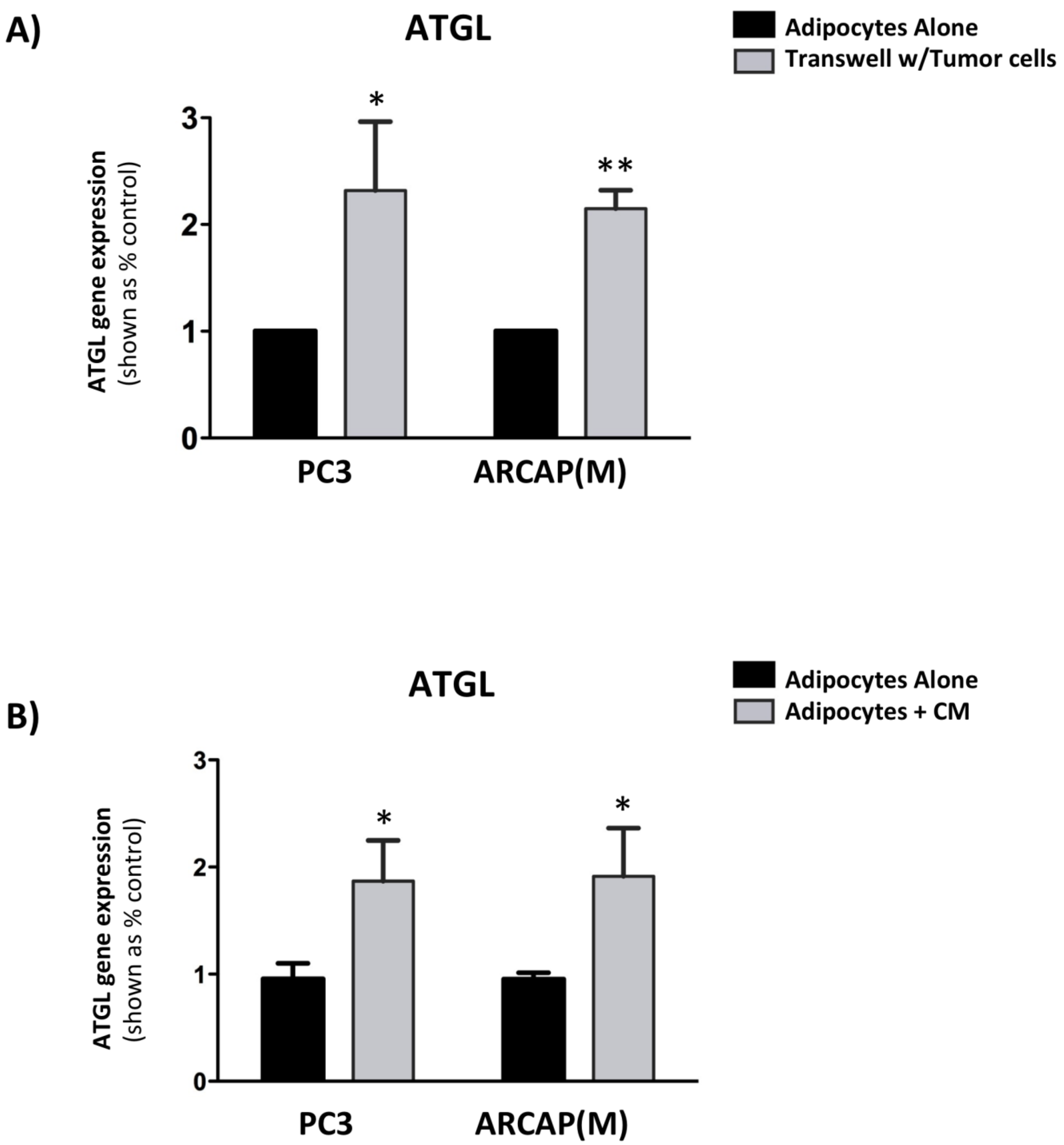

Supplementary Figure 9. Taqman RT-PCR analysis of *adipose triglyceride lipase (ATGL)* expression in adipocytes grown in transwell co-culture with PC3 or ARCaP(M) cells (**A**) or treated with PC3 or ARCaP(M) conditioned media (**B**). RT-PCR data are normalized to mouse *adiponectin* and shown relative to control. Results represent a mean of at least 3 independent experiments ± SD. Values * $p < 0.05$; ** $p < 0.01$ are considered statistically significant.

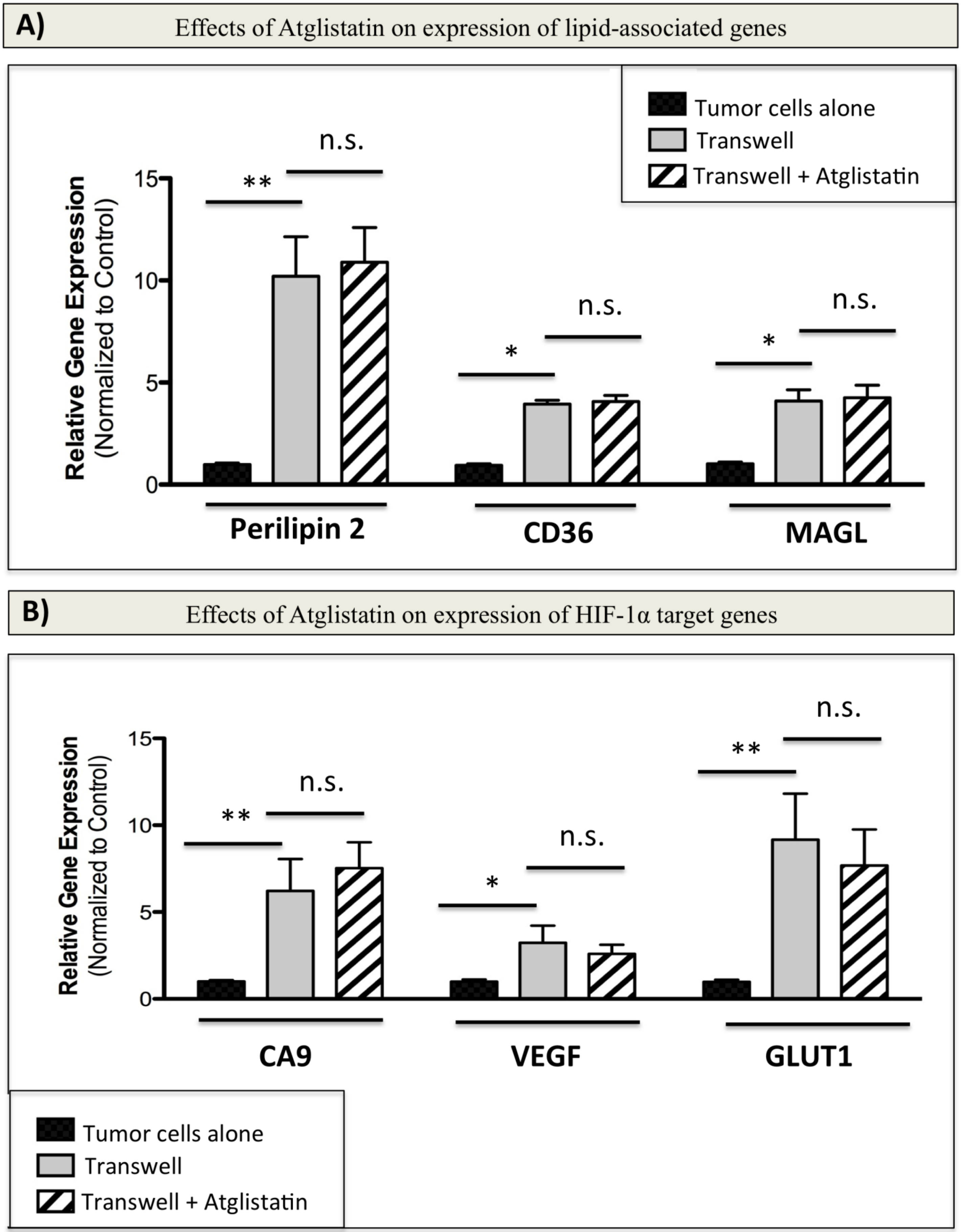
A)
Effects of Atglistatin on expression of lipid-associated genes
Tumor cells alone
Transwell
Transwell + Atglistatin
Relative Gene Expression
(Normalized to Control)
0
5
10
15
**
n.s.
*
n.s.
*
n.s.
Perilipin 2
CD36
MAGL
B)
Effects of Atglistatin on expression of HIF-1α target genes
Relative Gene Expression
(Normalized to Control)
0
5
10
15
**
n.s.
*
n.s.
**
n.s.
CA9
VEGF
GLUT1
Tumor cells alone
Transwell
Transwell + Atglistatin

Supplementary Figure 10. Inhibition of adipocyte lipolysis does not reduce the expression of lipid transporters and hypoxia-associated genes in tumor cells. Taqman RT-PCR analysis of *Perilipin 2, CD36,* and *MAGL*, expression (**A**) and *CA9, VEGF*, and *GLUT1* expression (**B**) in PC3 cells alone or in transwell co-culture with bone marrow adipocytes. Cells were cultured in the presence or absence of 10 µM Atglistatin. Data are normalized to *HPRT1* and shown as increase relative to control. Values * $p < 0.05$; ** $p < 0.01$ are considered statistically significant.

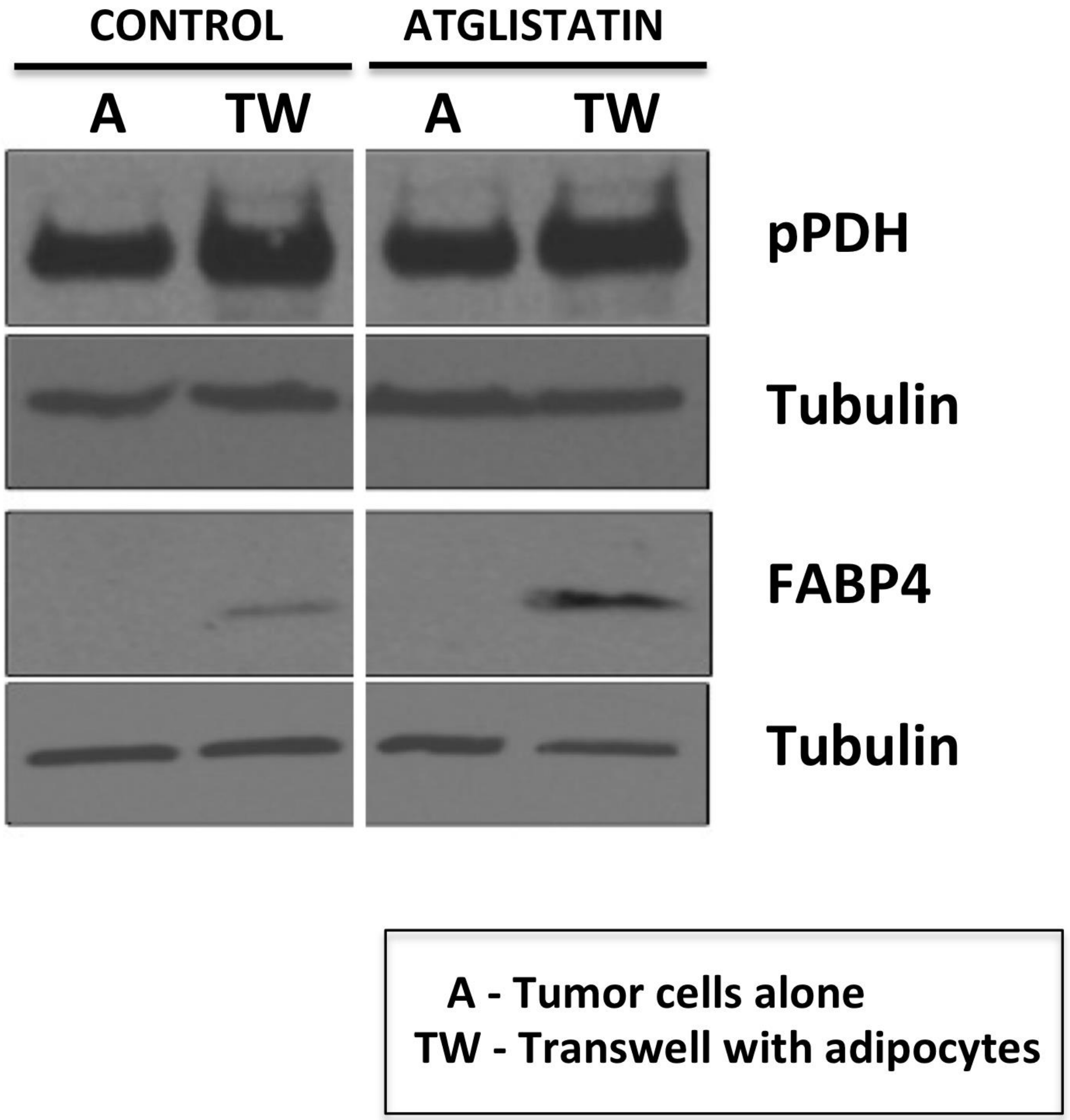

Supplementary Figure 11. Inhibition of lipolysis with Atglistatin enhances phosphorylation of PDH and increases expression of lipid transporter FABP4. Western blot analysis of phosphorylated PDH (top) and FABP4 (middle) in PC3 cells alone or in transwell co-culture in the absence or presence of 10 µM Atglistatin. Tubulin was used as a loading control (bottom).

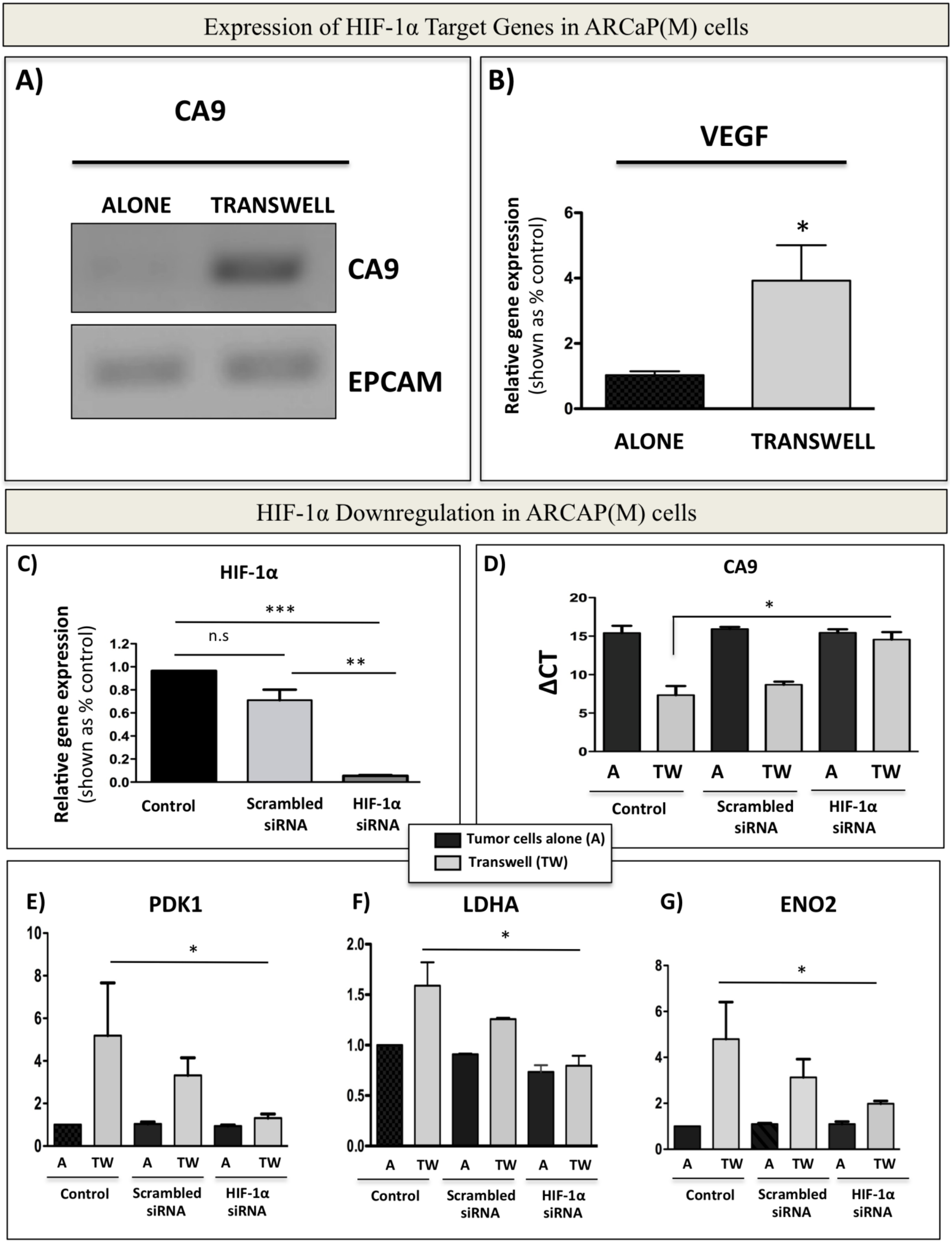

Expression of HIF-1α Target Genes in ARCaP(M) cells
A)
CA9
ALONE
TRANSWELL
CA9
EPCAM
B)
VEGF
Relative gene expression
(shown as % control)
6
4
2
0
*
ALONE
TRANSWELL
HIF-1α Downregulation in ARCAP(M) cells
C)
HIF-1α
Relative gene expression
(shown as % control)
1.2
1.0
0.8
0.6
0.4
0.2
0.0
***
n.s
**
Control
Scrambled siRNA
HIF-1α siRNA
D)
CA9
ΔCT
20
15
10
5
0
*
A
TW
A
TW
A
TW
Control
Scrambled siRNA
HIF-1α siRNA
Tumor cells alone (A)
Transwell (TW)
E)
PDK1
10
8
6
4
2
0
*
A
TW
A
TW
A
TW
Control
Scrambled siRNA
HIF-1α siRNA
F)
LDHA
2.0
1.5
1.0
0.5
0.0
*
A
TW
A
TW
A
TW
Control
Scrambled siRNA
HIF-1α siRNA
G)
ENO2
8
6
4
2
0
*
A
TW
A
TW
A
TW
Control
Scrambled siRNA
HIF-1α siRNA

Supplementary Figure 12. Adipocytes induce HIF-1α signaling in ARCaP(M) cells. A: *CA9* expression in ARCaP(M) cells alone or in transwell co-culture with bone marrow adipocytes as measured by semi-quantitative PCR. PCR products were resolved on 2% agarose gel. *EPCAM (CD326)* is used as a loading control (bottom). *CA9* expression is not detectable under control conditions. **B:** Taqman RT-PCR analysis of *VEGF* expression in ARCaP(M) alone or in tranwell co-culture with bone marrow adipocytes. **C:** mRNA levels of *HIF-1α* in ARCaP(M) cells grown under control conditions or treated with 10 μM scrambled siRNA, or 10 μM HIF-1α siRNA. **D:** Taqman RT-PCR analysis of the expression of HIF-1α target gene *CA9* to further confirm HIF-1α knockdown from cells grown in the presence or absence of adipocytes. Changes in CA9 mRNA levels are shown as ΔCT due to its low baseline expression under control conditions and upon HIF-1α knockdown in ARCAP(M) cells. **E-G:** Effect of HIF-1α knockdown on the mRNA expression of glycolysis associated genes: *PDK1* **(E),** *LDHA* **(F),** and *ENO2* **(G).** Data are the mean of analyses with 2 different siRNA constructs done in triplicate. Data are normalized to *HPRT1* and shown as increase relative to control. Values * $p < 0.05$; ** $p < 0.01$, and *** $p < 0.001$ are considered statistically significant.
